# Genome-Wide Identification of the β-Amylase (BAM) Gene Family in *Senna tora* and Seven Other Legumes: Characterization of *StoBAM6* as a Cold-Responsive Candidate Gene and a Potential Target of StoCBF2

**DOI:** 10.3390/plants15213407

**Published:** 2026-11-02

**Authors:** Zhao Feng, Jieyi Sun, Aman Wang, Pei Xi, Ruihua Lv, Gang Zhang

**Affiliations:** 1 College of Medical Technology, Shaanxi University of Chinese Medicine, Xianyang 712083, China; fengzhao2018@163.com (Z.F.);; 2 Key Laboratory for Research and Development of “Qin Medicine” of Shaanxi Administration of Traditional Chinese Medicine, Shaanxi University of Chinese Medicine, Xi’an 712046, China

**Keywords:** *Senna tora*, β-amylase, cold stress, starch metabolism

## Abstract

β-Amylases (BAMs) hydrolyze starch and contribute to abiotic stress acclimation, with AtBAM3 mediating most transitory starch degradation in *Arabidopsis*; however, legume BAM family evolution and the effect of whole-genome duplications remain unexplored. We identified 92 BAM genes across eight legume genomes and classified 111 BAM proteins (including *Arabidopsis* and rice references) into four groups, with subgroup II-B containing all AtBAM3 orthologs. In *S. tora*, *StoBAM6* and *StoBAM10* formed a collinear paralogous pair unlikely to derive from the Papilionoideae-specific duplication. Under 4 °C treatment, *StoBAM6* was induced within 1 h and stayed elevated through 24 h, whereas *StoBAM10* rose only transiently at 3 h. *StoBAM6* expression correlated positively with leaf β-amylase activity (*r* = 0.7557, *p* < 0.001) and negatively with starch content (*r* = −0.7343, *p* < 0.001), whereas *StoBAM10* showed no significant correlation with β-amylase activity (*r* = 0.7728, *p* = 0.75) or starch content (*r* = −0.0685, *p* = 0.77). *StoCBF2* shared *StoBAM6*’s cold-induced expression profile; yeast one-hybrid assays showed that StoCBF2 binds a *StoBAM6* promoter fragment harboring the DRE-core motif. These results indicate regulatory divergence after duplication, identify StoCBF2 as a candidate upstream regulator of *StoBAM6*, and provide a working model for cold-responsive starch metabolism in *S. tora*.

## 1. Introduction

Starch metabolism is crucial to plant growth and stress adaptation. During starch degradation, β-amylases (BAMs) hydrolyze glucan chains from their non-reducing ends to release maltose, which is exported from chloroplasts to the cytosol for further metabolism [1]. Under abiotic stress conditions such as low temperature or high salinity, maltose also acts as an osmoprotectant, helping to maintain the integrity of chloroplasts and cellular membranes [2,3]. BAM-mediated starch degradation also raises the levels of other soluble sugars such as glucose and sucrose, and localized starch degradation into sugars has been proposed to be important for the cold stress response, with the sugars providing osmoprotection [4]. BAM3 genes from pear and Amur grape have been reported to regulate soluble sugar accumulation together with ROS homeostasis or scavenging under cold stress [5,6]. Under osmotic stress, BAM1-dependent starch degradation in *Aarbidopsis* additionally supplies carbon for the synthesis of proline, another compatible solute [7], and abscisic acid regulates leaf starch degradation to support osmotic stress tolerance [8]. These dual roles in carbon distribution and stress protection make BAM genes important contributors to plant stress acclimation. During the evolution of angiosperms, the BAM gene family has undergone significant diversification. In *Arabidopsis thaliana*, nine BAM genes have been identified, and their functions are largely diversified [9]. AtBAM1 and AtBAM3 proteins have distinct cell-type-associated functions. AtBAM3 is responsible for the primary BAM activity in leaf mesophyll starch metabolism, and the downregulation of *AtBAM3* results in a starch-excess phenotype [10]. Under cold stress, *AtBAM3* is specifically induced, and its knockdown leads to decreased levels of maltose, glucose, fructose, and sucrose, increasing the sensitivity to cold stress [11]. *AtBAM1* is highly expressed in guard cells and plays a significant role in the rapid breakdown of guard cell starch to facilitate stomatal opening [12]. During osmotic stress, *AtBAM1* is upregulated and contributes to the degradation of starch, which in turn leads to the accumulation of sugar [8]. The AtBAM5 protein may participate in the degradation of starch released from the plastids of phloem sieve elements [13]. Other members of the family play completely different roles: AtBAM7 and AtBAM8 proteins have evolved into nuclear transcription factors containing a BZR1 domain [14,15]. The cold-responsive function of *AtBAM3*-like genes appears to be conserved in many plant species. In tomato (*Solanum lycopersicum*), *SlBAM3* promotes cold-induced starch degradation, and its overexpression enhances cold tolerance [16]. In tea (*Camellia sinensis*), low temperature likewise upregulates chloroplast-localized BAM genes, with downstream effects on starch hydrolysis and cold resistance [17]. CBF (C-repeat binding factor) transcription factors have been shown to regulate *AtBAM3*-like genes in trifoliate orange, kiwifruit, and pomegranate [18,19,20]. Evidence from these species links CBF-mediated regulation of *AtBAM3*-like genes to cold-induced starch catabolism in several angiosperm lineages, raising the question of whether this regulatory relationship is conserved in legumes.

Genome-wide studies of the BAM family and its role in cold response remain scarce in Leguminosae, even as evidence accumulates from other plant families. As one of the largest and agriculturally most important groups of flowering plants, legumes have undergone multiple whole-genome duplication (WGD) events. These duplications left behind genetic redundancy and subfunctionalization that complicate functional inference [21]. Comparing legume lineages with distinct WGD histories therefore provides a useful framework for investigating gene family evolution.

*Senna tora* (L.) Roxb. belongs to Caesalpinioideae, a subfamily that diverged from Papilionoideae before the papilionoid-specific WGD; its genome therefore lacks this duplication [22]. The mature seeds, known as Jue Ming Zi in traditional Chinese medicine, are rich in anthraquinones, including emodin and aurantio-obtusin [22]. Low temperature is an important abiotic stress that can restrict plant growth and disrupt carbon metabolism [2,3], but the molecular mechanisms linking cold responses with starch metabolism in *S. tora* remain poorly understood. Therefore, the distinct duplication history of *S. tora* makes it a useful caesalpinioid reference for comparing *BAM* gene retention and functional divergence with papilionoid legumes, while its medicinal importance provides a practical context for investigating cold-stress responses.

In this study, we conducted a genome-wide identification and comparative characterization of the *BAM* genes in *S. tora* and seven Papilionoideae species (*Glycine max*, *Medicago truncatula*, *Arachis hypogaea*, *Pisum sativum*, *Vigna angularis*, *Glycyrrhiza uralensis*, and *Astragalus membranaceus*), and based on these findings, we reconstructed the phylogenetic relationships, gene structures, conserved motifs, and syntenic contexts of the family across eight legume genomes. We then focused on the *S. tora* genes most closely related to *AtBAM3*, examining their expression patterns in different tissues and transcriptional responses to cold stress in relation to β-amylase activity and starch content dynamics. Finally, we investigated the upstream regulatory mechanism using a yeast one-hybrid assay to test binding of a cold-responsive CBF transcription factor to the candidate gene promoter. Our results provide insights into BAM family evolution in legumes and identify *StoBAM6* as a cold-responsive candidate gene associated with starch metabolism in *S. tora*, providing a basis for future functional studies of cold acclimation in this medicinal species.

## 2. Results

### 2.1. Identification and Characterization of BAM Family Members in S. tora and Related Legume Species

**Figure 1:**
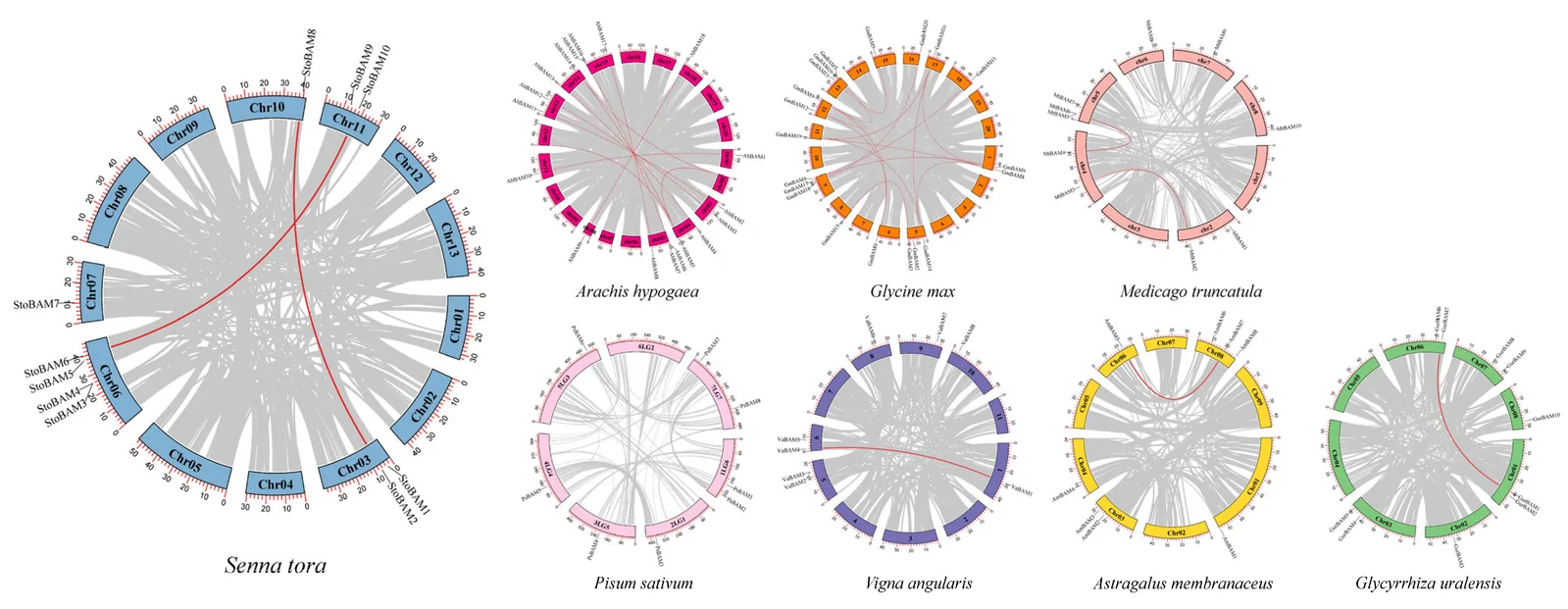
Chromosomal localization and syntenic relationship of *BAM* genes in eight legume genomes. Circos plots show collinear gene pairs within each of the eight legume genomes: *S. tora* (enlarged on the left as the focal species), *A. hypogaea*, *G. max*, *M. truncatula*, *P. sativum*, *V. angularis*, *A. membranaceus*, and *G. uralensis*. Chromosomes are shown as colored arcs along the outer ring with chromosome IDs labeled, and *BAM* gene positions are indicated outside the outer ring. Grey lines in the inner area show genome-wide collinear gene pairs identified by MCScanX, and red lines highlight *BAM* gene pairs classified as WGD/segmental duplicates. The red line in the *S. tora* plot connects *StoBAM6* and *StoBAM10*, which form a collinear paralogous pair examined in detail in this study.

Using HMMER searches followed by domain verification, we identified 92 *BAM* genes across eight legume species: 10 in *S. tora*, 20 in soybean, 18 in peanut, 10 each in *M. truncatula* and *G. uralensis*, and 8 each in *A. membranaceus*, garden pea and *V. angularis*. The 18 peanut *BAM* genes matched the number reported by Shi et al. (2025) [23]. Soybean contained approximately 2.5 times as many *BAM* genes as *A. membranaceus*, garden pea and *V. angularis*, a pattern consistent with the additional whole-genome duplication in the *Glycine* lineage [21,24]. *S. tora*, a member of Caesalpinioideae, contains ten *BAM* genes, a number similar to those found in several diploid papilionoid species examined here. MCScanX classified 15 *BAM* gene pairs in soybean, 11 in peanut, two each in *M. truncatula* and *S. tora*, and one each in *G. uralensis*, *V. angularis*, and *A. membranaceus* as WGD/segmental duplicates. One tandemly duplicated *BAM* gene pair was additionally identified in soybean, whereas no WGD/segmental or tandemly duplicated *BAM* gene pairs were detected in garden pea. Notably, one of the two WGD/segmental pairs in *S. tora* corresponds to *StoBAM6* and *StoBAM10*, the two *AtBAM3*-like genes identified in this study (Figure 1).

### 2.2. Phylogenetic Analysis of BAM Family Members in S. tora and Related Legume Species

**Figure 2:**
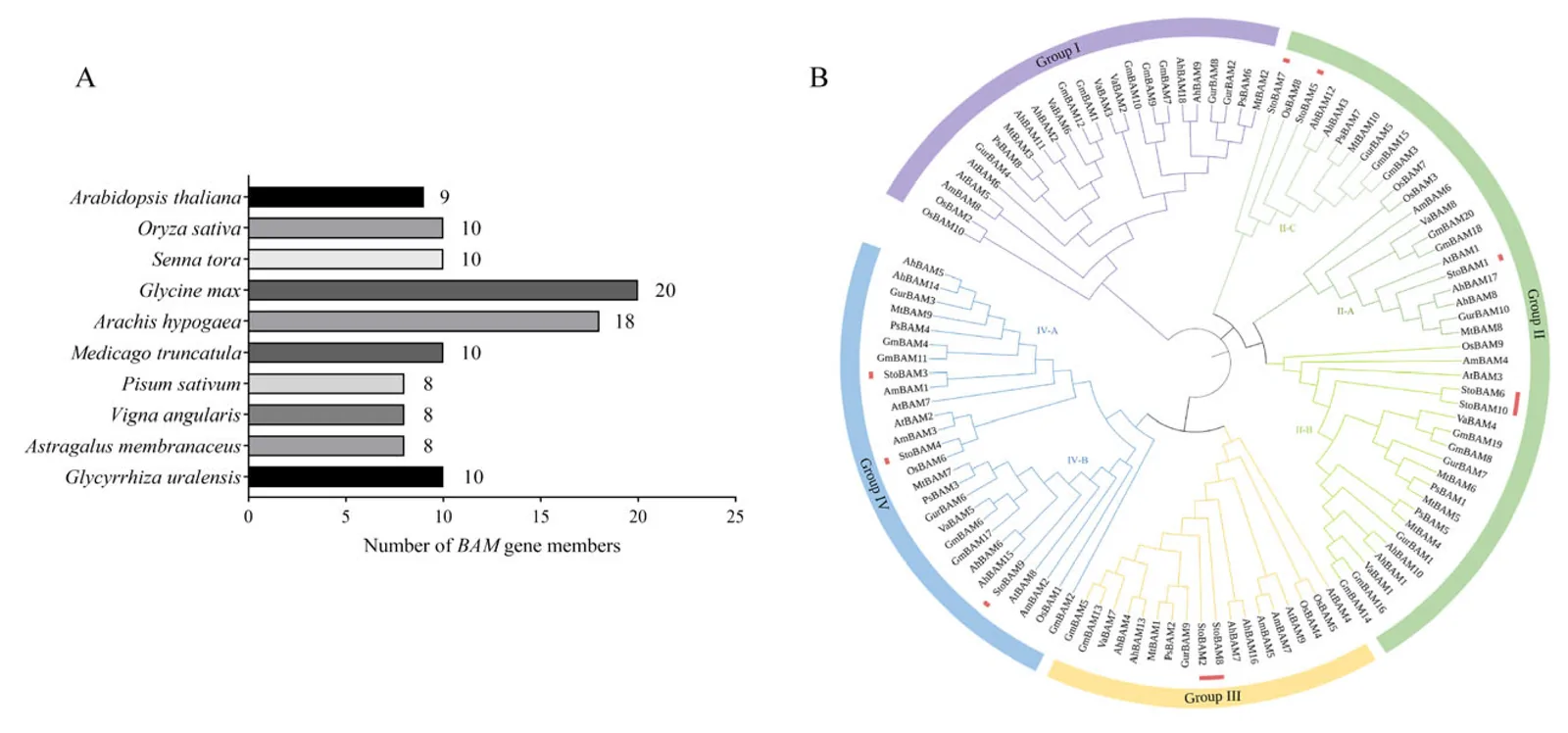
*BAM* gene family distribution and phylogenetic classification across *Arabidopsis*, rice, and eight legume species. (**A**) Number of BAM members in *A. thaliana*, *O. sativa*, *S. tora*, *G. max*, *A. hypogaea*, *M. truncatula*, *P. sativum*, *V. angularis*, *A. membranaceus*, and *G. uralensis*. Numbers to the right of each bar indicate the total count per species. (**B**) Neighbor-Joining tree of 111 BAM proteins constructed in MEGA 12 with 1000 bootstrap replicates, classifying members into four groups (I–IV); Group II is subdivided into II-A, II-B, and II-C, and Group IV into IV-A and IV-B. Red bars on the outer ring mark the ten S. tora BAM members. The names of species were abbreviated as follows: At, *A. thaliana*; Os, *O. sativa*; Sto, *S. tora*; Gm, *G. max*; Ah, *A. hypogaea*; Mt, *M. truncatula*; Ps, *P. sativum*; Va, *V. angularis*; Am, *A. membranaceus*; Gur, *G. uralensis*.

A neighbor-joining tree constructed from 92 legume BAM proteins, 9 AtBAM proteins, and 10 OsBAM proteins separated these sequences into four groups (Groups I-IV), containing 24, 42, 18, and 27 members, respectively. Group II is subdivided into subgroups II-A, II-B, and II-C, and Group IV into IV-A and IV-B (Figure 2). Within subgroup II-B, StoBAM6 and StoBAM10 cluster with AtBAM3, a chloroplast-localized β-amylase that mediates transitory starch degradation in *Arabidopsis* [10]. This phylogenetic placement suggests that StoBAM6 and StoBAM10 are the most likely AtBAM3 orthologs in *S. tora*. The rice protein OsBAM9 also falls in this subgroup, suggesting that this AtBAM3-like lineage is conserved between monocots and legumes.

### 2.3. Physicochemical Properties and Predicted Subcellular Localization of BAM Proteins in S. tora and Related Legume Species

The 111 BAM proteins differ widely in size and physicochemical properties. Length ranges from 222 aa (GmBAM2) to 1332 aa (AmBAM2), molecular weight from 25.22 to 149.34 kDa, and pI from 4.60 to 10.10. This variation is unevenly partitioned among the four groups. Group II is the most conserved; within subgroup II-B, most members are similar in protein length (mostly 540–550 aa) and predicted to localize to chloroplasts or chloroplast thylakoid membranes, although a small number of members, such as PsBAM5, showed different localization predictions. Group IV sits at the other extreme, containing both the smallest BAMs in the dataset (GmBAM2, 222 aa; StoBAM4, 263 aa) and the largest (AhBAM5, 1144 aa; AmBAM2, 1332 aa), thus spanning almost the full size range. Groups I and III are intermediate. Their pI distributions are broader and their predicted localizations more heterogeneous, with several members assigned to the extracellular space rather than the chloroplast (Table S1).

### 2.4. Gene Structure Analysis of BAM Family Members in S. tora and Related Legume Species

**Figure 3:**
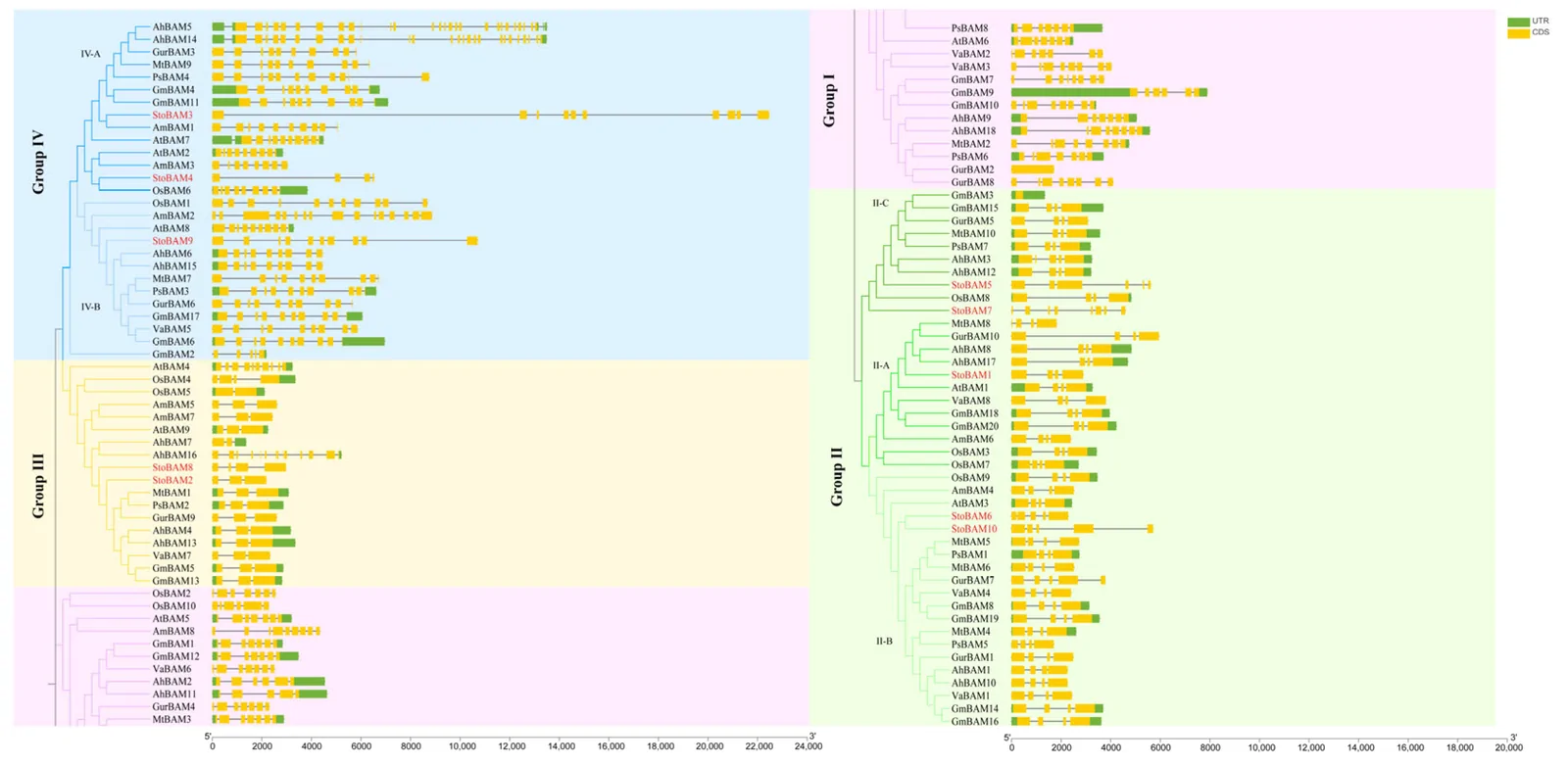
Gene structures of 111 *BAM* genes from *Arabidopsis*, rice, and eight legume species. Gene structures were drawn based on annotated GFF files using TBtools-II. Yellow boxes indicate coding sequences (CDS), while green boxes indicate untranslated regions (UTR). Introns are shown as black lines. The phylogenetic tree on the left of each panel groups members by clade, with background colors marking the four major groups (I–IV).

Exon number differs markedly among the four groups. Groups I and IV are exon-rich: 83.3% of Group I members (20/24) carry 7–9 exons, and 81.5% of Group IV members (22/27) fall within 8–11. The other groups are far more compact. All 12 members of subgroup II-A have exactly four exons, and the four-exon configuration likewise dominates subgroup II-B (17/20; 85%), with a few members carrying five. In subgroup II-C, four exons remain the most common arrangement (6/10; 60%), though the remaining members are structurally more variable. Group III is the most reduced of all, with 83.3% of members (15/18) adopting a three-exon structure (Figure 3).

A parallel subgroup-associated pattern was also observed in signature exon length and arrangement. Group I members generally contain five core exons in a fixed order: 239, 209, 263, 164, and 398 nt. Subgroups II-A and II-B commonly share a conserved 210 nt–110 nt core pair, and in some II-B members a fifth exon has been acquired through 3′ intron insertion. The 110 nt core expands to 113 nt in subgroup II-C, where exon splitting and intron loss are also more frequent. Group III carries a single diagnostic central exon of 482 or 485 nt. The two Group IV subgroups diverge more sharply: subgroup IV-A is built from conserved modular exons (77, 164, 194, 203, 218, 219, and 260 nt) with a characteristic 28 nt micro-exon near the 5′ end, whereas several IV-B members carry giant exons exceeding 1100 nt that arose through intron fusion, alongside locally conserved exons of 114 and 167 nt.

### 2.5. Conserved Motif Analysis of BAM Family Members in S. tora and Related Legume Species

**Figure 4:**
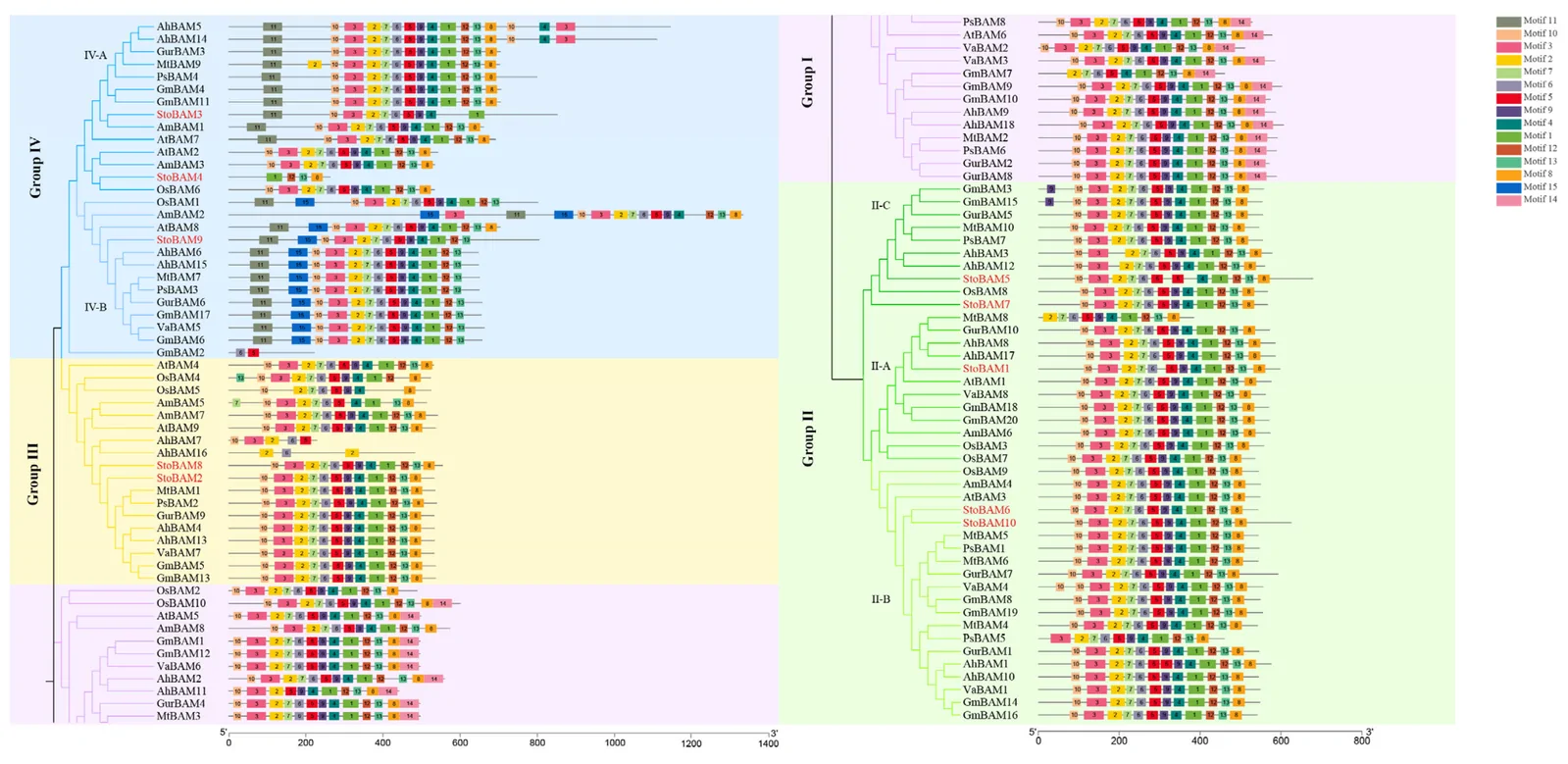
Conserved motif architecture of 111 BAM proteins from eight legume species and two reference genomes. Conserved motifs were identified using MEME with the maximum number of motifs set to 15. Each colored box represents a distinct motif (Motif 1–Motif 15) as shown in the legend. Background colors mark the four major groups (I–IV). Scale bars at the bottom indicate protein length in amino acids; the protein-length scale differs between the left (0–1400 aa) and right (0–800 aa) panels.

MEME analysis identified 15 conserved motifs (Motifs 1 to 15) in the 111 BAM proteins (Figure 4). Compared with the gene structure described above, the motif composition was much more conserved. A core arrangement of Motifs 10-3-2-7-6-5-9-4-1-12-13-8 occurred in most members of all four groups and in every species examined, and this region probably represents the functional domain of the protein. Differences between groups were nonetheless evident in the peripheral motifs. Most Group I proteins ended in Motif 14, which was absent from the other three groups. Groups II and III retained the core arrangement and added no group-specific motif. Group IV differed mainly at the N-terminus, where most members began with Motif 11. Within this group, subgroup IV-B placed Motif 15 directly after Motif 11, and this Motif 11–Motif 15 pair set IV-B apart from IV-A. A few proteins departed from these patterns. AmBAM2 carried duplicated copies of Motifs 15 and 3 and was correspondingly longer than the rest, whereas GmBAM2 and AhBAM16 were severely truncated and retained few of the core motifs.

### 2.6. Cis-Regulatory Element Analysis of StoBAM Promoters

**Figure 5:**
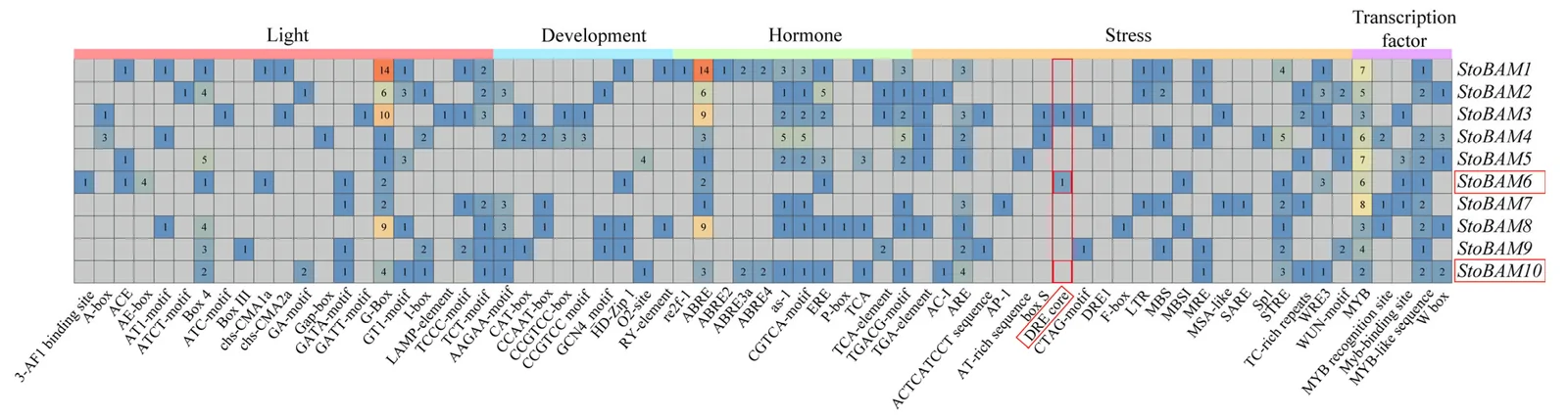
Distribution of putative cis-regulatory elements in the 2000-bp upstream regions of the ten *StoBAM* genes. Cis-regulatory elements were predicted using PlantCARE from the 2000-bp upstream sequence of each *StoBAM* gene. Each colored cell indicates the number of predicted elements of a particular type in the corresponding promoter, with abundance represented by the colour gradient; gray cells indicate that the element was not detected. The elements are grouped into five functional categories: light-responsive, development-related, hormone-responsive, stress-responsive, and transcription factor binding sites as indicated by the coloured bars above the heatmap.

Analysis of the 2-kb upstream regions of all ten *StoBAM* genes was carried out using PlantCARE, and a total of 69 cis-regulatory element types were identified. These elements were classified into five categories: light-responsive (21 types), stress-responsive (22 types), hormone-responsive (12 types), growth and development-related (9 types), and transcription factor binding sites (5 types). Among them, light-responsive elements including the G-box were the most widely distributed. Particularly high copy numbers were observed in *StoBAM1*, *StoBAM3*, and *StoBAM8* (14, 10, and 9 copies, respectively). For hormone-responsive elements, ABRE was present in most members, and *StoBAM1* contained as many as 14 copies. CGTCA-motif and TGACG-motif, which are both MeJA-responsive, were found in at least eight genes, and ERE and TCA-element were also commonly detected. In addition, MYB recognition sequences were observed in all ten promoters (Figure 5).

Several putative elements previously associated with abiotic-stress response were also predicted, including the anaerobic response element (ARE), the drought-associated MYB binding site (MBS), the low-temperature responsive element (LTR), defense-related TC-rich repeats, and the wound-responsive WUN-motif. A putative DRE-core motif (CCGAC) was detected in the *StoBAM6* promoter but not in the *StoBAM10* promoter (highlighted in Figure 5). This difference represents a candidate cis-regulatory feature that may be associated with the divergent cold-responsive expression patterns of the two paralogs. Previous promoter surveys of BAM families in other species have likewise reported LTR, ABRE and MBS element [20,25]. The co-occurrence of LTR elements with high-frequency ABRE and MBS elements is therefore consistent with the transcriptional regulation of specific StoBAM genes under cold stress conditions.

### 2.7. Expression of StoBAM Genes Across Tissues and Developmental Stages

**Figure 6:**
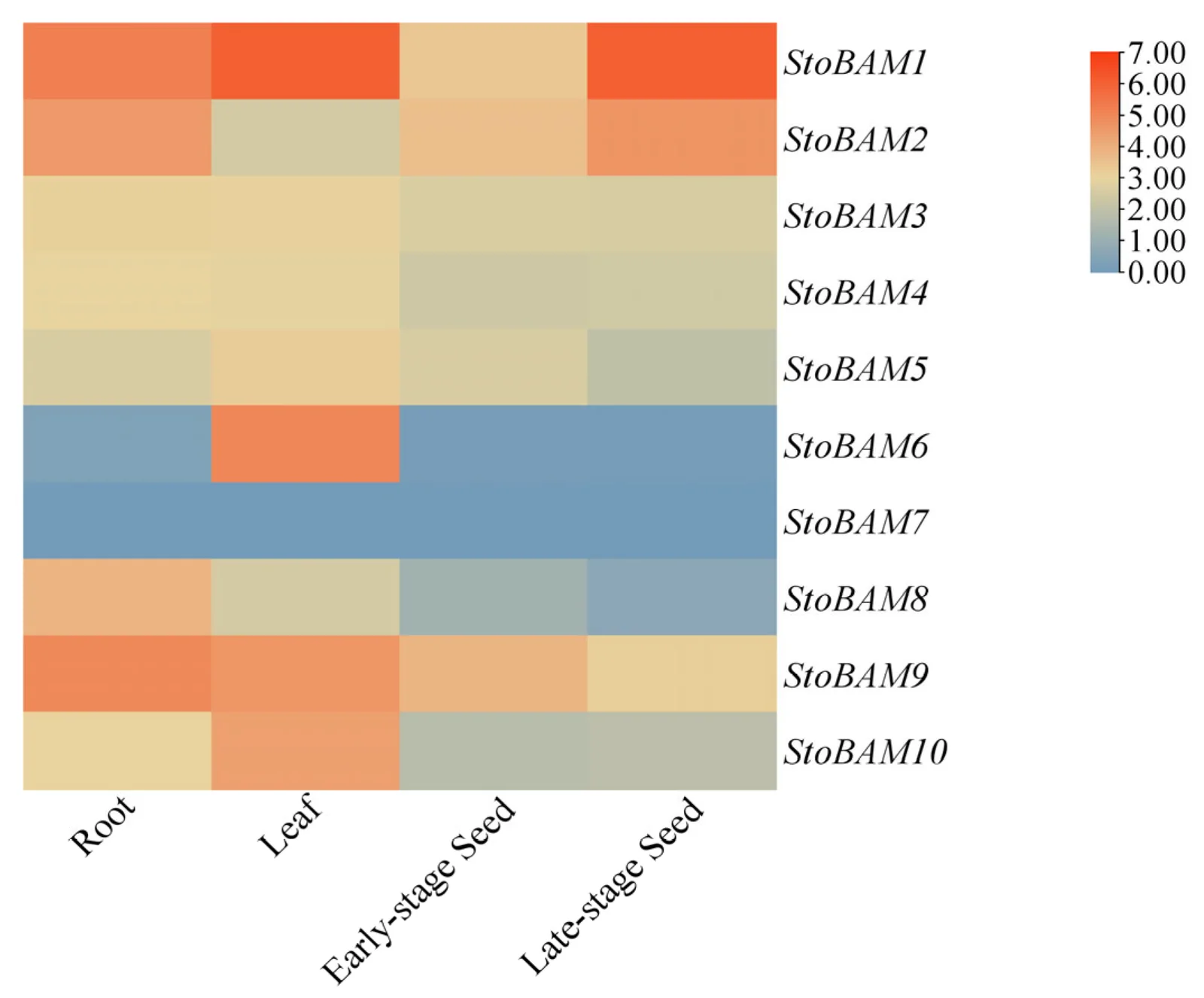
Tissue-specific expression profiles of the ten *StoBAM* genes in *Senna tora*. The heatmap shows relative expression levels of *StoBAM1*-*StoBAM10* in root, leaf, early-stage seed, and late-stage seed samples based on publicly available RNA-seq data from study accession SRP159435. Expression values are shown as log2 (TPM + 1), with color intensity scaled according to the legend on the right. *StoBAM6* shows clear leaf-preferential expression among the four tissues examined, supporting its selection for subsequent analysis of the leaf cold response.

To investigate the tissue-specific expression patterns of the ten *StoBAM* genes, publicly available RNA-seq data from root, leaf, immature seed, and late-stage seed samples were analyzed, and transcript abundance was visualized as log2 (TPM + 1) (Figure 6). The ten genes exhibited distinct expression patterns among the four tissues. *StoBAM1* showed relatively high transcript abundance in all four tissues, particularly in leaf and late-stage seed samples. *StoBAM2* and *StoBAM9* were also expressed in all four tissues, whereas *StoBAM3*, *StoBAM4* and *StoBAM5* showed comparatively low transcript abundance. *StoBAM7* remained at a very low level in all four tissues. *StoBAM8* showed higher transcript abundance in root than in leaf or seed samples. *StoBAM6* and *StoBAM10* showed their highest transcript abundance in leaf samples. *StoBAM6* was expressed at a low level in roots and both seed stages, whereas *StoBAM10* showed lower transcript abundance in both seed stages than in root and leaf samples.

### 2.8. StoBAM6 Rapidly Responded to Cold Treatment in S. tora Leaves

**Figure 7:**
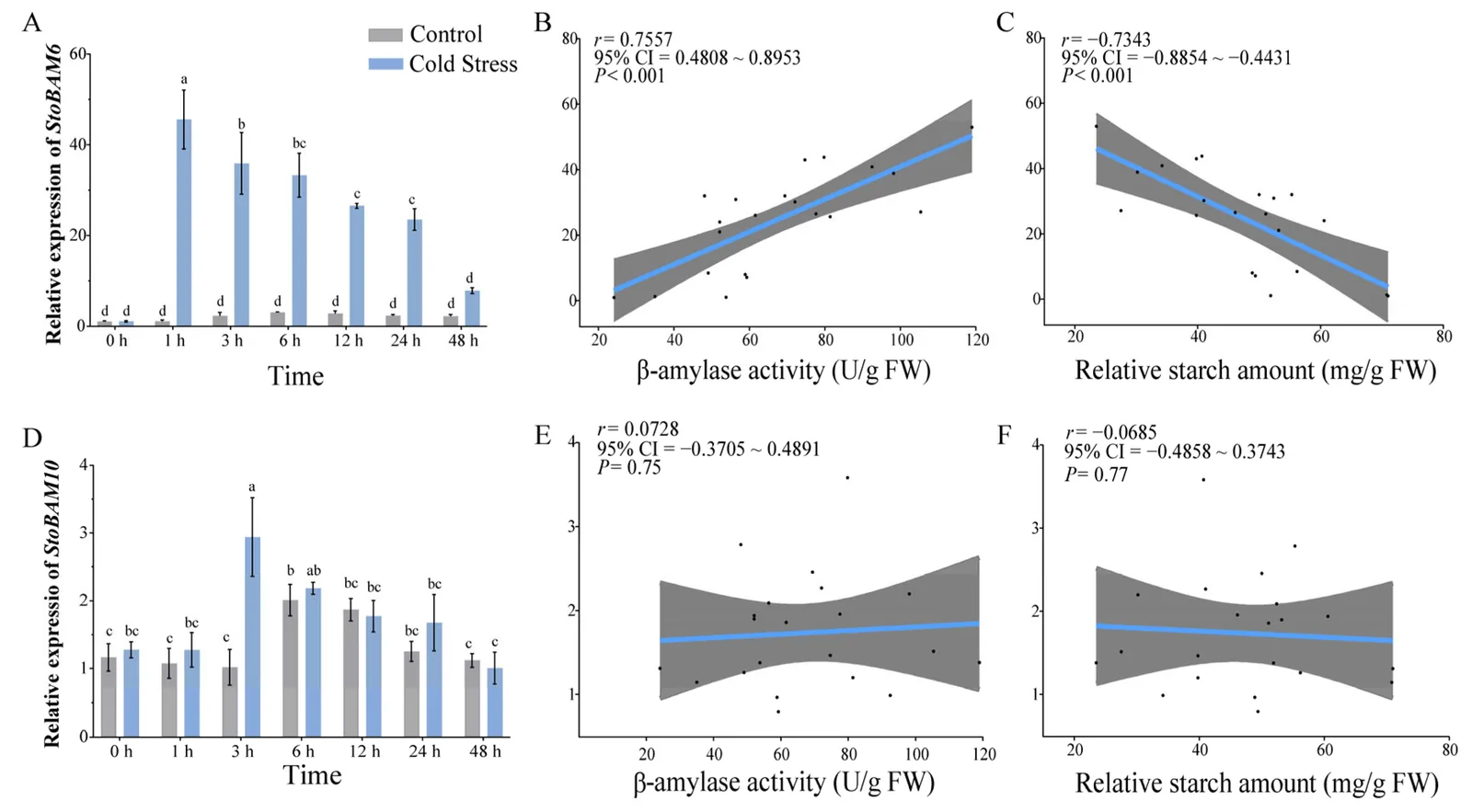
Cold-induced expression and physiological correlations of *StoBAM6* and *StoBAM10* in *S. tora* leaves. (**A**) Relative expression of *StoBAM6* under cold stress (4 °C, blue) and control (25 °C temperature, gray) at 0, 1, 3, 6, 12, 24, and 48 h. (**B**) Pearson correlation between *StoBAM6* transcript abundance and leaf β-amylase activity. (**C**) Pearson correlation between *StoBAM6* transcript abundance and leaf starch content. (**D**) Relative expression of *StoBAM10* under cold and control conditions. (**E**) Pearson correlation between *StoBAM10* transcript abundance and leaf β-amylase activity. (**F**) Pearson correlation between *StoBAM10* transcript abundance and leaf starch content. For bar charts, data are presented as mean ± SD from three biological replicates. The effects of temperature treatment, sampling time, and their interaction were evaluated using two-way ANOVA. Different lowercase letters indicate significant differences among the 14 treatment-time combinations according to Duncan’s multiple range test at *p* < 0.05. For the correlation plots, only cold-treated samples were included. Each point represents paired gene-expression and physiological measurements obtained from the same biological replicate, with 21 paired observations included in each analysis (seven sampling time points × three biological replicates). Blue lines indicate the linear regression fit and gray shaded areas represent the 95% confidence interval. Pearson’s r values, 95% confidence intervals, and *p* values are shown in each plot.

We measured the transcript levels of *StoBAM6* and *StoBAM10* in *S. tora* leaves over a 48-h cold-treatment course. Control samples were excluded because the correlation analysis was designed to test whether, among the cold-treated samples, changes in *StoBAM6* transcript abundance track changes in β-amylase activity and starch content during the time course. Control plants (25 °C) represent a different treatment condition, and pooling them with cold-treated samples would combine two treatment groups, so that the correlation could reflect the treatment difference rather than the relationship between gene expression and physiology. The effect of cold relative to control on gene expression was assessed separately by two-way ANOVA (Figure 7A,D). *StoBAM6* was significantly induced at 1 h and remained significantly up-regulated at subsequent sampling points through 24 h, whereas no significant difference from the corresponding control was detected at 48 h (Figure 7A). *StoBAM10* was significantly induced only at 3 h (Figure 7D); levels at all other time points were not significantly different from those of the control. Across the 4 °C treatment time course, *StoBAM6* transcript abundance was positively correlated with leaf β-amylase activity (*r* = 0.7557, *p* < 0.001) and negatively correlated with starch content (r = −0.7343, *p* < 0.001) based on 21 paired observations from the same biological samples (Figure 7B,C). In contrast, *StoBAM10* transcript abundance was not significantly correlated with either β-amylase activity (*r* = 0.0728, *p* = 0.75) or starch content (*r* = −0.0685, *p* = 0.77) (Figure 7E,F). During the cold-treatment time course, *StoBAM6* transcript abundance was associated with leaf β-amylase activity and starch content, whereas no significant associations were detected for *StoBAM10*.

### 2.9. Gene Structure and Local Synteny of AtBAM3-like Homologs in Subgroup II-B

AtBAM3, also designated BMY8, is the chloroplast β-amylase whose transcription responds most strongly to low temperature, and plants in which it is silenced fail to accumulate maltose during cold shock and suffer greater photosystem II damage upon freezing [11]. This dependence is not confined to *Arabidopsis*. A cold-induced ortholog in Actinidia arguta, AaBAM3.1, raised freezing tolerance when overexpressed in both kiwifruit and *Arabidopsis*, and protein-DNA assays confirmed that the cold-responsive factor AaCBF4 binds and directly activates it [19]. The grapevine genes *VvBAM1* and *VvBAM3* followed a comparable pattern, with transcript levels rising as temperature fell, and tomato plants expressing *VvBAM1* degraded more starch, retained higher glucose and sucrose, and withstood cold better than the wild type [26]. Cold-responsive members of the same branch were later recovered from genome-wide surveys in the legume white clover [25] and in pomegranate [20]. Subgroup II-B, the orthologous group of AtBAM3, was therefore examined in detail.

**Figure 8:**
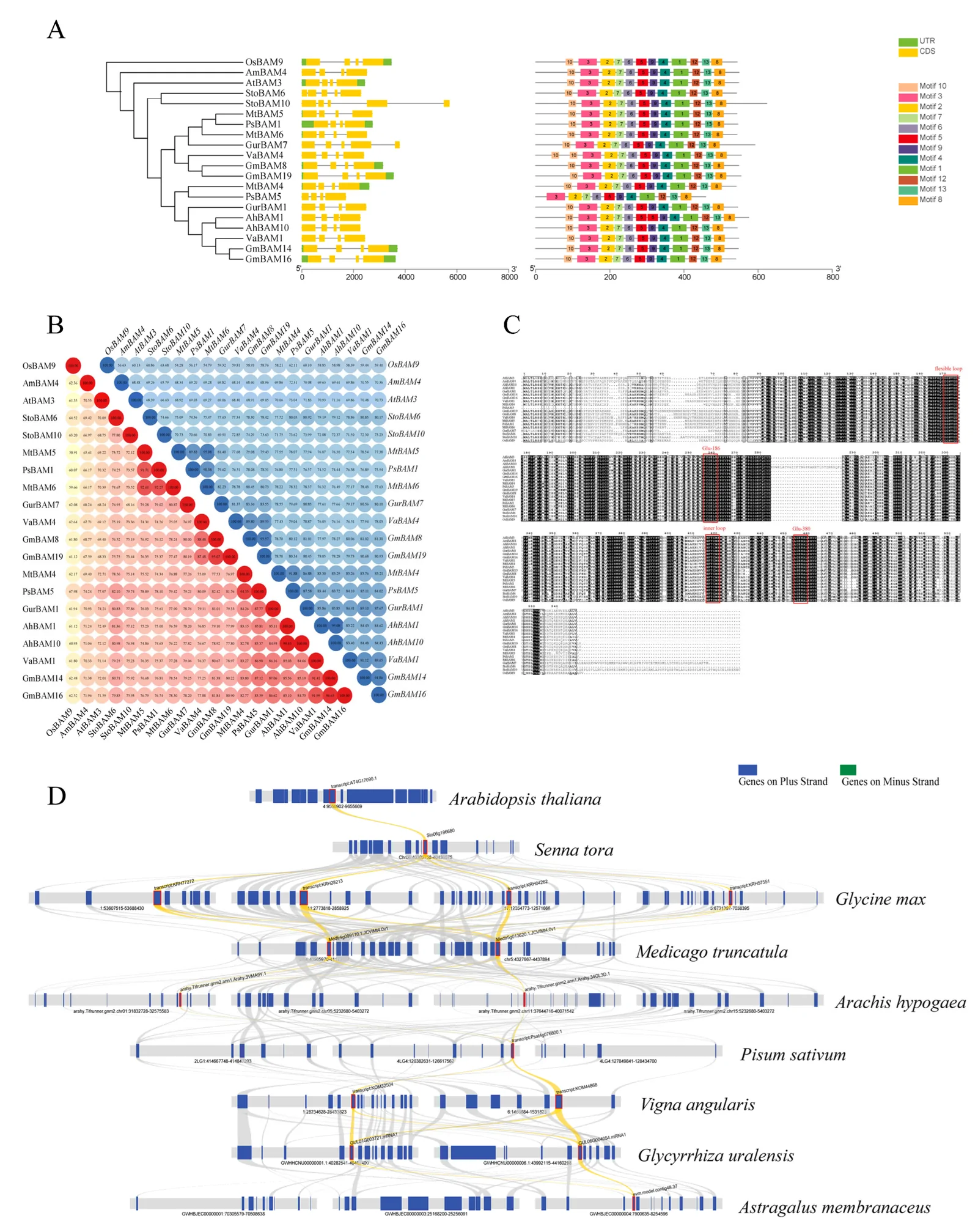
Structural and sequence comparison of subgroup II-B BAM proteins (AtBAM3 orthologs) across legumes and reference species. (**A**) Exon–intron structures (**left**) and conserved motif architecture (**right**) of 20 subgroup II-B BAM members, including AtBAM3 from *A. thaliana*, OsBAM9 from *O. sativa* as the rice member that roots the subgroup tree, and 18 BAM3 orthologs from the eight legume species examined. Members are arranged according to a Neighbor-Joining phylogeny shown on the far left. In the motif panel, colored boxes represent Motif 1–Motif 15 as defined in Figure 4. In the gene structure panel, yellow boxes indicate CDS and green boxes indicate UTR. Scale bars at the bottom indicate gene length in base pairs (**left**) and protein length in amino acids (**right**). (**B**) Pairwise amino acid and CDS identity matrix of the 20 BAM proteins; identity values of proteins (%) are shown in the lower-left triangle (red circles) and the similarity of CDSs visualized by color intensity in the upper-right triangle (blue circles). (**C**) Multiple sequence alignment of the 20 BAM proteins. Identical and conserved residues are shaded in black and grey, respectively. Red boxes highlight four conserved features characteristic of BAM proteins: the flexible loop, the inner loop, and the two active site residues Glu-186 and Glu-380, with residue numbering referring to AtBAM3. Species abbreviations are as in Figure 1. (**D**) Local microsynteny between *StoBAM6* (Sto06g196680) and its corresponding orthologs in *A. thaliana* and seven other legume species. Genomic regions of each species are represented by gray bars; blue boxes indicate genes on the plus strand and green boxes indicate genes on the minus strand. Red boxes mark *StoBAM6* and its syntenic orthologs in each species. Yellow lines highlight the collinear gene pairs centered on StoBAM6, whereas gray lines denote other collinear gene pairs within these regions.

Subgroup II-B comprises 20 proteins: 18 putative AtBAM3-like proteins from the eight legume species, together with AtBAM3 from *A. thaliana* and OsBAM9 from *O. sativa*. The 18 legume proteins were StoBAM6, StoBAM10, GmBAM8, GmBAM14, GmBAM16, GmBAM19, AhBAM1, AhBAM10, MtBAM4, MtBAM5, MtBAM6, AmBAM4, GurBAM1, GurBAM7, PsBAM1, PsBAM5, VaBAM1, and VaBAM4 (Figure 8). The genes encoding most subgroup II-B members contained four exons, whereas *StoBAM6*, *StoBAM10*, and *GurBAM7* contained five exons. The corresponding proteins shared the conserved motif arrangement Motif 10-3-2-7-6-5-9-4-1-12-13-8, except PsBAM5, which lacks Motif 10 at its 5′ end (Figure 8A). Most legume proteins in subgroup II-B showed approximately 70% amino acid identity to AtBAM3, including AhBAM1 (72.49%), PsBAM1 (70.32%), and StoBAM6 (70.09%). CDS identity was similarly conserved, ranging from 68% to 71% (Figure 8B). Multiple sequence alignment showed that all subgroup II-B proteins retained the four characteristic BAM features: the flexible loop, the inner loop, and the two active site residues Glu-186 and Glu-380 (Figure 8C) [27].

**Figure 9:**
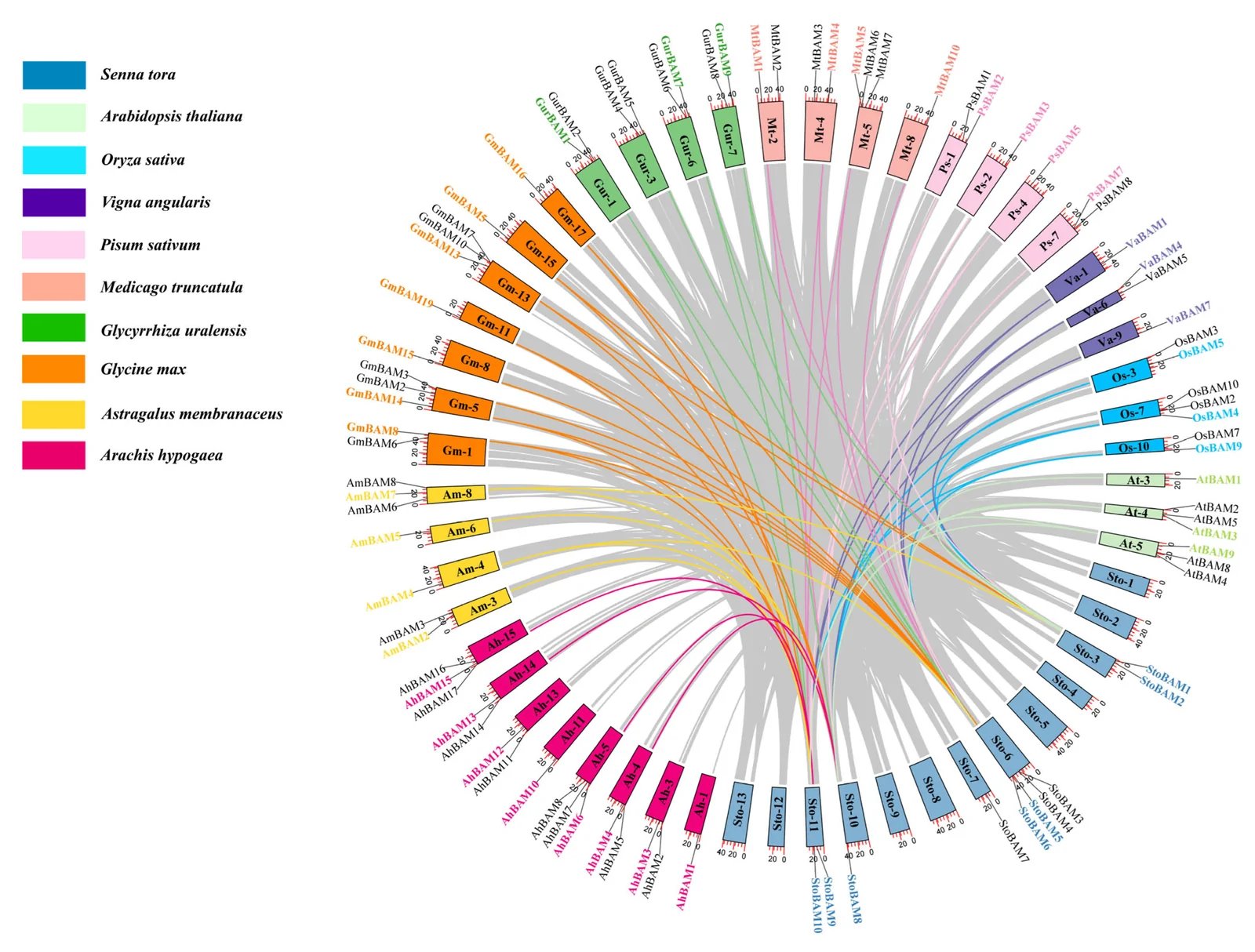
Synteny relationships of *BAM* genes between *S. tora* and nine comparison species. The nine comparison species are *A. thaliana*, *O. sativa*, *M. truncatula*, *G. max*, *A. hypogaea*, *P. sativum*, *V. angularis*, *A. membranaceus*, and *G. uralensis*. Chromosomes of different species are shown in different colors (as indicated in the legend), and the scale on the outer ring represents chromosome length (Mb). Gray lines represent genome-wide collinear gene pairs between species, whereas colored lines highlight the syntenic relationships involving *S. tora BAM* genes.

*StoBAM6* (chromosome 6) and *StoBAM10* (chromosome 11) each showed syntenic relationships with 16 *BAM* genes from the comparison species (Figure 9). Within the *S. tora* genome, *StoBAM6* and *StoBAM10* formed a collinear paralogous pair, which MCScanX classified as a WGD/segmental duplicate (Figure 1 and Figure 9). Because *S. tora* belongs to Caesalpinioideae, which diverged from Papilionoideae before the Papilionoideae-specific WGD [21,22], a duplicate pair in its genome cannot have arisen from that event. However, the current synteny evidence cannot distinguish whether the pair was retained from an older duplication in Caesalpinioideae or arose from a later duplication in the *S. tora* lineage. The chromosomal neighborhood of *StoBAM6* showed conserved collinearity with homologous regions in *A. thaliana* and the seven other legume species examined. The number of BAM homologs within these syntenic regions varied among species, with four in *G. max* and two each in *A. hypogaea*, *M. truncatula*, *V. angularis*, and *G. uralensis* (Figure 8D). These homologs were located on different chromosomes within each genome, consistent with their classification as WGD/segmental rather than tandem duplicates. The *StoBAM6*–*StoBAM10* pair therefore provides a useful system for investigating post-duplication regulatory divergence.

### 2.10. Identification of StoCBF2 as a Candidate Upstream Regulator of StoBAM6

**Figure 10:**
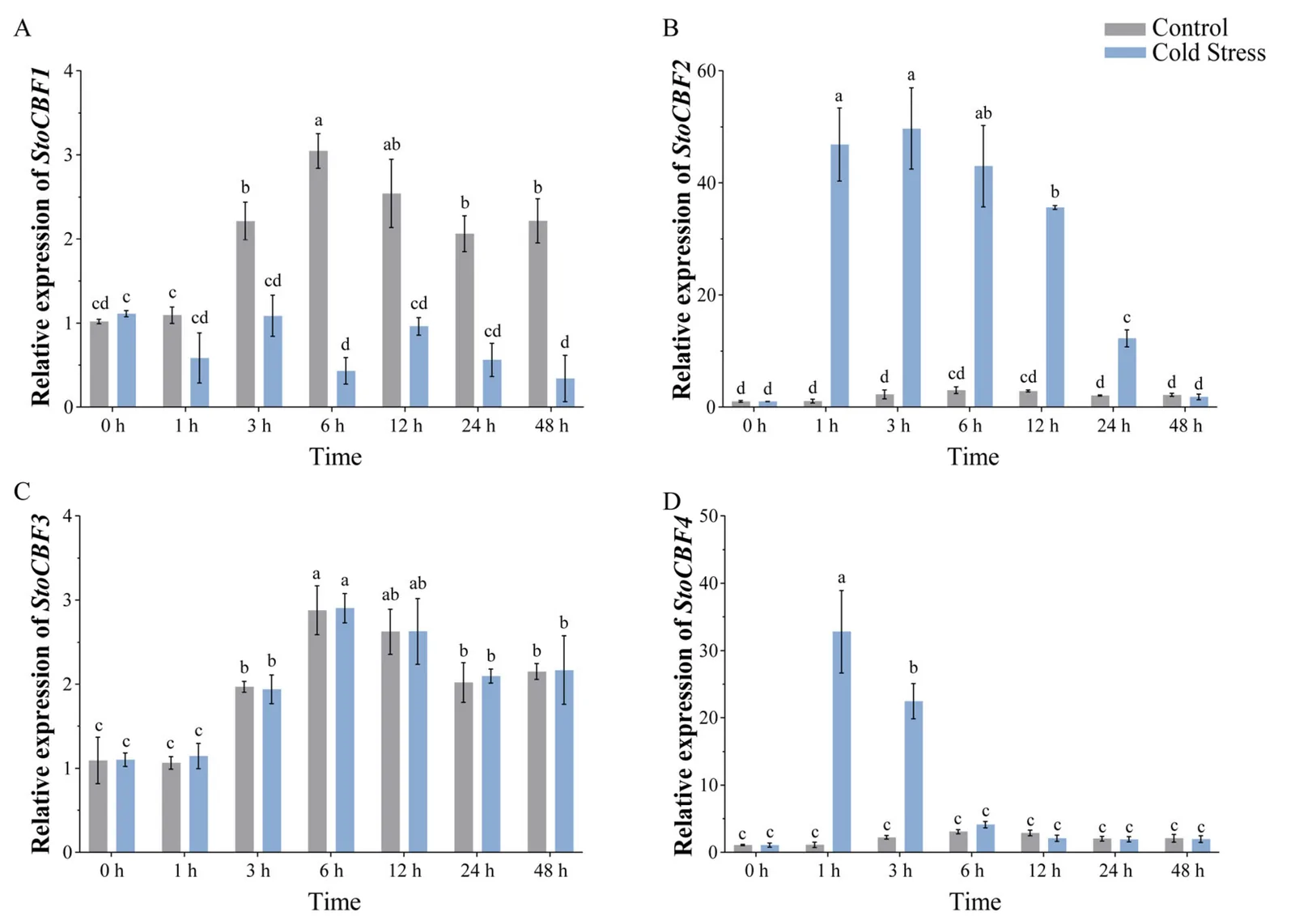
Cold-induced expression profiles of the four *StoCBF* genes in *Senna tora* leaves. Relative expression levels of *StoCBF1* (**A**), *StoCBF2* (**B**), *StoCBF3* (**C**), and *StoCBF4* (**D**) under cold stress (4 °C, blue bars) and control conditions (25 °C temperature, gray bars) at 0, 1, 3, 6, 12, 24, and 48 h post-treatment. Data are presented as mean ± SD from three biological replicates. The effects of temperature treatment, sampling time, and their interaction were evaluated using two-way ANOVA. Different lowercase letters indicate significant differences among the 14 treatment–time combinations according to Duncan’s multiple range test at *p* < 0.05. Note that the Y-axis scales differ between panels (0–4 for *StoCBF1* and *StoCBF3*; 0–60 for *StoCBF2*; 0–50 for *StoCBF4*) due to the markedly different expression levels and induction magnitudes of the four genes.

Having established that *StoBAM6* is rapidly and persistently induced by cold, we next sought to identify a candidate upstream transcription factor potentially associated with this response. Because one DRE-core (CCGAC) motif was detected in the *StoBAM6* promoter (Figure 5), we focused on the CBF/DREB transcription factor family. Four *CBF* genes were identified in the *S. tora* genome and designated *StoCBF1*–*StoCBF4* (Figure 10). In a neighbor-joining tree, StoCBF2 (Sto07g214110) was placed in Group III together with AtCBF1, AtCBF2, AtCBF3 and AtCBF4 (Figure S1), members of the *Arabidopsis* CBF/DREB1 subfamily. Quantitative RT-PCR analysis of all four *StoCBF* genes under 4 °C treatment showed two distinct response patterns. *StoCBF2* was significantly upregulated from 1 h onward and remained elevated through 24 h, with transcript levels returning to control values by 48 h (Figure 10B). *StoCBF4* responded only transiently, showing significant induction at 1 h and 3 h but no significant difference from the control at 6 h and later (Figure 10D). *StoCBF1* and *StoCBF3* were not significantly induced under cold stress at any time point. Compared with the transient induction of *StoCBF4*, the sustained induction of *StoCBF2* from 1 to 24 h more closely resembled the expression pattern of *StoBAM6*, which was induced by 1 h and remained elevated throughout the 24-h time course. *StoCBF2* was therefore selected for further analysis, and its encoded protein was evaluated as a candidate upstream regulator of *StoBAM6* under cold stress.

**Figure 11:**
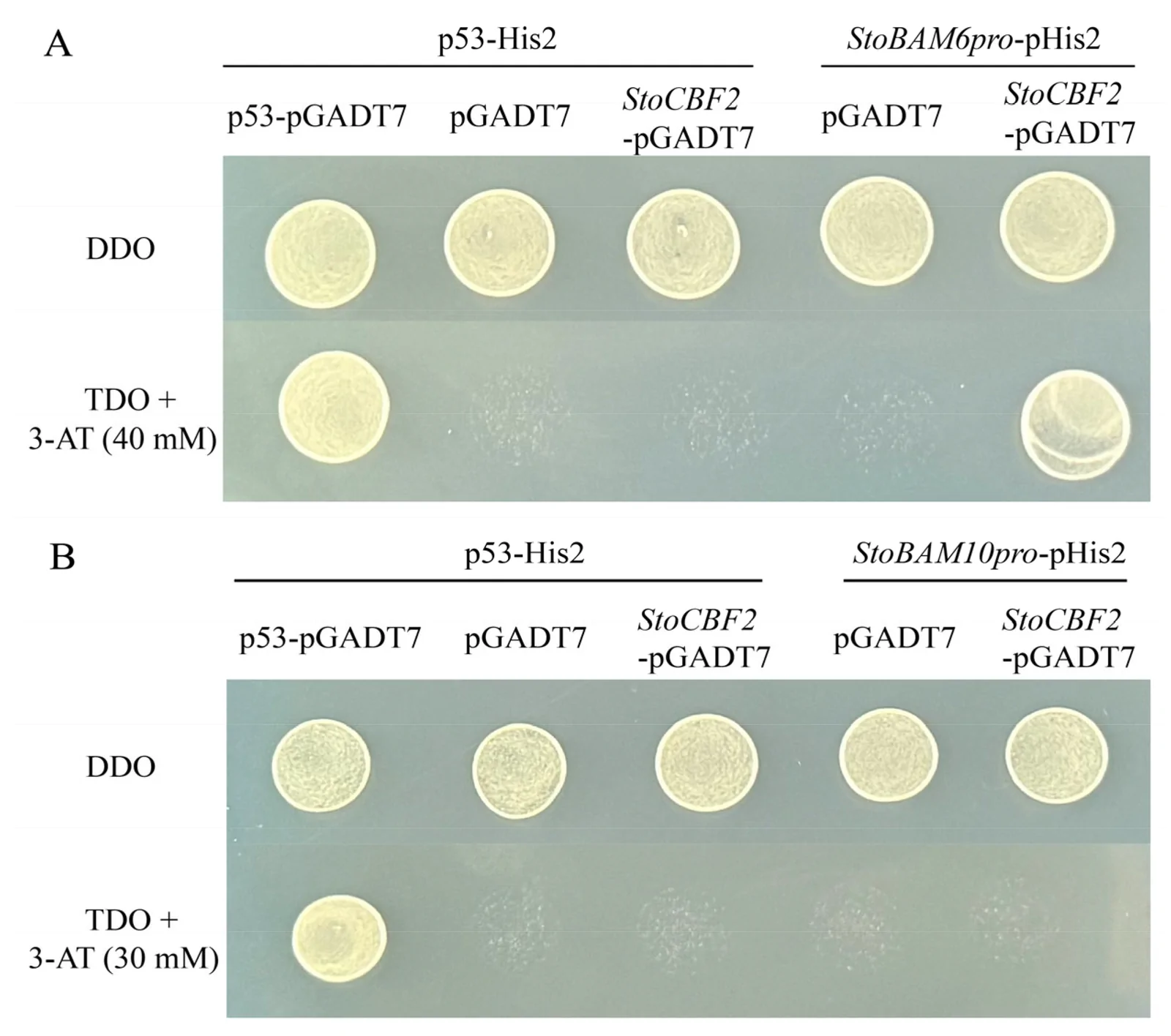
Yeast one-hybrid (Y1H) assays testing the binding of StoCBF2 to the *StoBAM6* and *StoBAM10* promoters. (**A**) Interaction between StoCBF2 and the *StoBAM6* promoter. (**B**) Interaction between StoCBF2 and the *StoBAM10* promoter. *StoCBF2*-pGADT7 was co-transformed with *StoBAM6pro*-pHis2 (**A**) or *StoBAM10pro*-pHis2 (**B**) into yeast cells, which were grown on double-dropout (DDO, SD/-Leu/-Trp) and triple-dropout (TDO, SD/-Leu/-Trp/-His) medium supplemented with 3-AT. The p53-pGADT7 + p53-His2 combination served as the positive control, whereas p53-His2 + pGADT7, p53-His2 + *StoCBF2*-pGADT7, and the corresponding promoter-pHis2 + pGADT7 combinations served as negative controls. 3-AT was added at 40 mM (**A**) and 30 mM (**B**). Growth on TDO + 3-AT medium indicates an interaction between the protein and the promoter fragment in yeast.

To test whether StoCBF2 could bind the *StoBAM6* and *StoBAM10* promoters, we performed a yeast one-hybrid (Y1H) assay using separate promoter fragments of about 2 kb of *StoBAM6* (1978 bp) and *StoBAM10* (1906 bp) as bait and the full-length *StoCBF2* coding sequence as prey. The 3-AT titration showed that 40 mM and 30 mM were sufficient to suppress the background HIS3 activity of the *StoBAM6* and *StoBAM10* promoter constructs, respectively, because yeast carrying only *StoBAM6pro*-pHis2 or *StoBAM10pro*-pHis2 failed to grow on SD/-Trp/-His medium containing the corresponding 3-AT concentration (Figures S2 and S3). Yeast co-transformed with *StoBAM6pro*-pHis2 + *StoCBF2*-pGADT7 grew on SD/-Leu/-Trp/-His with 40 mM 3-AT, as did the positive control (p53-His2 + p53-pGADT7) (Figure 11A), while *StoBAM10pro*-pHis2 + *StoCBF2*-pGADT7 did not grow on SD/-Leu/-Trp/-His with 30 mM 3-AT (Figure 11B). Negative controls (p53-His2 + pGADT7; p53-His2 + *StoCBF2*-pGADT7; *StoBAM6pro*/*StoBAM10pro*-pHis2 + pGADT7) were viable on SD/-Leu/-Trp but not on the triple-dropout medium. The Y1H results showed that StoCBF2 can bind the *StoBAM6* promoter fragment harboring the DRE-core motif in yeast, whereas it did not bind the *StoBAM10* promoter fragment, which lacks this motif, under the condition tested. These findings support StoCBF2 as a candidate upstream regulator of *StoBAM6* under cold stress.

## 3. Discussion

The *BAM* gene family plays important roles in starch metabolism and plant responses to environmental stress, and variation in family size among species has been widely reported [20,23,26]. Across the eight legume genomes examined here, we identified 92 *BAM* genes, ranging from eight members in *A. membranaceus*, *P. sativum*, and *V. angularis* to 20 in *G. max*. The approximately 2.5-fold higher number in soybean is consistent with its lineage-specific whole-genome duplication [24]. MCScanX classified 15 *BAM* gene pairs in soybean and 11 in peanut as WGD/segmental duplicates, whereas only one tandemly duplicated pair was identified in soybean. No WGD/segmental or tandemly duplicated *BAM* gene pairs were detected in *P. sativum* in the present analysis. *S. tora*, which diverged before the Papilionoid-specific WGD, contained ten *BAM* genes and two WGD/segmental duplicate pairs, numbers comparable to those in several diploid papilionoid species examined here. Collectively, these results suggest that WGD/segmental duplication contributed more strongly than tandem duplication to the expansion of the *BAM* gene family in the legumes examined [20,28].

Phylogenetic analysis classified 111 BAM proteins, including 92 legume BAM proteins together with nine AtBAMs and ten OsBAMs reference proteins, into four major groups (I–IV), with Groups II and IV further divided into subgroups. This classification was broadly consistent with those reported for *Arabidopsis* [9], white clover [25], pomegranate [20], jujube [29], and upland cotton [30], suggesting that the major BAM lineages were established before the diversification of the legume species examined here. Members within each group generally shared similar structural characteristics. Subgroup II-B, for instance, showed a conserved motif arrangement containing the catalytic residues Glu-186 and Glu-380, and most of its members were predicted to localize to chloroplasts. By contrast, the four major groups differed substantially in gene structure, protein length, and predicted subcellular localization. Similar patterns of within-group conservation and between-group divergence have been reported in several other plant species [9,25,29,30].

The cold-responsive function of AtBAM3 orthologs has been well documented as an example of functional conservation within the BAM family. *AtBAM3* was shown to be strongly induced by low temperature in *Arabidopsis* leaves [2], and the encoded protein was later confirmed to localize to chloroplasts [10,31]. It has also been reported that *bam3* knockout plants accumulate excess starch in leaves and show impaired growth and photosynthetic performance under cold stress [10,32]. Based on these studies, AtBAM3 has been considered the major β-amylase responsible for chloroplast transitory starch degradation, particularly under low-temperature conditions [9,33]. The maltose released by this hydrolysis acts as an osmoprotectant and membrane stabilizer under cold conditions [3]. The same cold-inductive mode has been reported across different species. *PtrBAM1* from trifoliate orange carries a CBF-recognizing element in its promoter and overexpression enhances cold tolerance [18]; the expression of *PbrBAM3* from birchleaf pear (*Pyrus betulaefolia*) is upregulated by cold, dehydration, and ABA [5]; *AaBAM3.1* from kiwifruit is directly activated by *AaCBF4* [19]; *SlBAM3* from tomato is induced via the JA-MYC2 signaling module, with CRISPR/Cas9 knockout impairing cold-induced starch catabolism [16,34]; and *VaBAM3* from wild grape shares high structural similarity with *AtBAM3*, is cold-induced, and its overexpression elevates cold tolerance [6]. In rice, *OsMYB30* negatively regulates cold tolerance by interacting with a JAZ protein to repress *BAM* gene expression and reduce maltose accumulation, further demonstrating that *BAM* genes occupy a central node in cold signaling networks [35].

Within *S. tora*, StoBAM6 and StoBAM10 proteins clustered with AtBAM3 in subgroup II-B. The corresponding *StoBAM6* and *StoBAM10* genes each contained five exons, whereas their encoded proteins shared a conserved motif arrangement, the two catalytic glutamate residues, and predicted chloroplast localization. Both proteins showed approximately 70% amino acid identity to AtBAM3. Multiple sequence alignment further confirmed that StoBAM6 and StoBAM10 retained the flexible loop, inner loop, and catalytic residues Glu-186 and Glu-380 characteristic of BAM proteins [27]. Transcriptome profiling showed that *StoBAM6* and *StoBAM10* were preferentially expressed in leaves relative to roots and seeds, consistent with the established role of *AtBAM3* in leaf starch metabolism [9,10]. Synteny analysis placed the *StoBAM6* (chromosome 6) and *StoBAM10* (chromosome 11) loci within genomic regions showing conserved collinear relationships with homologous regions in the seven other legume species examined. Within the *S. tora* genome, the two genes constituted one of only two WGD/segmental duplicate pairs identified in the BAM family (Figure 1). Because *S. tora* diverged from Papilionoideae before the Papilionoid-specific WGD [21,22], this pair is unlikely to have originated from that event. It may instead have been retained from an older duplication in Caesalpinioideae or arisen through a later duplication in the *S. tora* lineage. Together with their phylogenetic placement and conserved protein architecture, these findings support *StoBAM6* and *StoBAM10* as putative *AtBAM3*-like genes in *S. tora*.

The activity of plastidic BAMs is also shaped by the chemical environment of the chloroplast. In *Arabidopsis*, *BAM1* and *BAM3* both show optimum activity at pH 6.0–6.5 in vivo, but *BAM1* is more active than *BAM3* at high pH and is more thermostable, consistent with *BAM1* acting during the day and under osmotic stress and *BAM3* acting at night and under cold stress [32]. This difference is relevant because the pH of the chloroplast stroma rises from about 7 in the dark to about 7.8–8.0 in the light [36]. *BAM1* is, in addition, redox-regulated by thioredoxins [7,37]. *StoBAM6* and *StoBAM10* are *AtBAM3*-like, but their pH and thermal properties have not been tested. In the present study, pH was not manipulated, and enzyme activity was measured under the buffer conditions of the assay kit; the influence of pH on *StoBAM6* and *StoBAM10* activity therefore remains to be determined.

β-Amylase functions in the starch and sucrose metabolism pathway and has also been linked to plant stress responses. In our cold treatment experiments, the two *S. tora* AtBAM3 paralogs behaved very differently. *StoBAM6* was significantly induced at 1 h of 4 °C exposure and remained significantly upregulated throughout 24 h, whereas its expression was not significantly different from the corresponding control at 48 h. *StoBAM10*, in contrast, was only transiently induced at 3 h, and its expression at the other time points was not significantly different from that of the control. The fast and sustained induction of *StoBAM6* is similar to what has been reported for cold-responsive *AtBAM3* orthologs in trifoliate orange, kiwifruit, pear, tomato, and wild grape [5,6,18,19,34], while the weak and transient induction of *StoBAM10* is similar to the reduced cold response that has been reported for some *AtBAM3* paralogs in polyploid legume genomes. For example, in the allotetraploid white clover (*Trifolium repens*), three *AtBAM3* orthologs (*TrBAM04*, *TrBAM09*, and *TrBAM10*) have been identified, but only *TrBAM04* and *TrBAM10* carry DRE-core motifs in their promoters, and the three paralogs were shown to have clearly different cold-induction profiles [25]. As described above, *StoBAM6* and *StoBAM10* form a duplicated gene pair that is unlikely to have originated from the Papilionoideae-specific WGD. Their divergent cold-responsive expression patterns are consistent with regulatory divergence following duplication. Distinct expression patterns and promoter responses have also been reported for a duplicated response regulator gene pair in *Populus deltoides* [38]. Subfunctionalization involves the partitioning of ancestral functions between duplicated genes [39], but whether the divergence observed here represents this process remains to be determined. Under this scenario, *StoBAM6* may have retained the cold-responsive role of *AtBAM3*, while *StoBAM10* may have evolved a more restricted function.

The transient induction of *StoBAM10* (only at 3 h) contrasts with the sustained induction of *StoBAM6*. One possible explanation lies in promoter architecture: *StoBAM10* lacks the DRE-core motif present in *StoBAM6* and did not bind StoCBF2 in yeast, so it may not be sustained by the CBF pathway, and its short-lived induction may instead depend on other cis-elements, such as the MYB recognition sites found in all ten *StoBAM* promoters (Section 2.6). In white clover, paralogs of *AtBAM3* likewise differ in DRE-core content and show clearly different cold-induction profiles [25], and a duplicated gene pair in *Populus deltoides* shows divergent expression and promoter responses [38]. The 3 h induction of *StoBAM10* also coincides with the transient induction of *StoCBF4* at 1 and 3 h (Figure 10D), although a link remains to be tested. In addition, feedback regulation or acclimation after the initial cold response may contribute, as both *StoBAM6* and *StoCBF2* returned to control levels by 48 h. Because control plants were sampled at each time point, the transient pattern is unlikely to reflect circadian effects, but a finer sampling interval would be needed to resolve the kinetics. These explanations are hypotheses that require functional testing.

At the physiological level, *StoBAM6* transcript abundance was positively correlated with leaf β-amylase activity (*r* = 0.7557, *p* < 0.001) and negatively correlated with starch content (*r* = −0.7343, *p* < 0.001) across 21 paired observations from the 4 °C treatment time course (Figure 7). In contrast, neither relationship was significant for *StoBAM10*. Similar associations among gene expression, enzyme activity, and starch content have also been reported for *VaBAM3* in wild grape and *StBAM1* in potato [6,40]. Taken together, these results identify *StoBAM6* as a strong cold-responsive candidate whose expression is associated with β-amylase activity and starch content during the cold-treatment time course in *S. tora* leaves.

One copy of the CCGAC motif, which represents the DRE-core sequence recognized by CBF/DREB transcription factors through their AP2/ERF domain [41], was identified in the *StoBAM6* promoter. In our yeast one-hybrid assay, StoCBF2 bound the *StoBAM6* promoter fragment containing the DRE-core motif. Because the fragment is about 2 kb long, whether StoCBF2 recognizes the CCGAC motif itself, rather than other sequences within the fragment, remains to be determined. Similar CBF-BAM regulatory connections have been reported in several other species. Peng et al. [18] reported that *PtrBAM1* in trifoliate orange contains a CBF-recognition element and was identified as a member of the CBF regulon. In kiwifruit, AaCBF4 was shown to bind to the *AaBAM3.1* promoter and to activate its expression under cold stress, which contributed to enhanced cold tolerance [19]. A similar Y1H interaction between PgCBF7 and the *PgBAM4* promoter was also reported in pomegranate [20]. In wild grape, however, VaWRKY65 was found to regulate *VaBAM3* independently of the CBF pathway, suggesting that BAM activation in cold response can occur through CBF-independent routes as well [42]. Future work should include site-directed mutagenesis of the DRE-core motif combined with Y1H or EMSA, dual-luciferase reporter assays in planta, and overexpression or knockout/silencing of *StoCBF2* and *StoBAM6* to test their effects on starch turnover and cold tolerance.

Although *StoCBF4* was also induced under cold stress, its transient expression pattern differed from the sustained induction of *StoBAM6*, leading us to prioritize StoCBF2 as a candidate upstream regulator. Together with the phylogenetic, expression, and physiological evidence, the Y1H results support StoCBF2 as a candidate upstream transcription factor capable of binding the *StoBAM6* promoter fragment in yeast. Because Y1H is a heterologous system, it cannot exclude indirect interactions mediated by yeast proteins, and the binding therefore needs to be confirmed in planta. All cold-treatment analyses in this study were performed on leaf tissue. Whether the StoCBF2–*StoBAM6* regulatory module operates similarly in other tissues, such as roots or seeds, remains to be investigated. Further genetic and in planta promoter analyses are required to determine whether StoCBF2 directly regulates *StoBAM6* during cold stress and whether this putative regulatory relationship contributes to cold tolerance in *S. tora*. Because *Arabidopsis bam3* mutants accumulate less anthocyanin during prolonged cold [43], future studies could also examine whether *StoBAM6*-dependent starch turnover influences the accumulation of medicinal metabolites in *S. tora*. Our experiments used a constant 4 °C treatment, and the responses of *StoBAM6* and *StoCBF2* to fluctuating temperatures, such as repeated cold–warm cycles, were not tested. Sustained induction of *StoBAM6* and the associated starch degradation may help to accumulate protective sugars in the cold, but under fluctuating temperatures repeated activation could carry costs, for example, by reducing the carbon reserves available for growth, and cold-induced starch breakdown has adverse effects in some systems, such as cold-induced sweetening of potato tubers [40]. Conversely, *Arabidopsis bam3* mutants, in which *BAM3*-mediated starch breakdown is impaired, accumulate excess starch and show impaired growth and photosynthetic performance under cold [10,32], suggesting that a balance in starch turnover is important. Whether the StoCBF2–StoBAM6 module shows similar trade-offs in *S. tora* under fluctuating temperatures should be examined in future work.

## 4. Materials and Methods

### 4.1. Plant Materials and Treatment Methods

Seeds of *S. tora* were collected from the Medicinal Plant Garden of Shaanxi University of Chinese Medicine. Seeds of uniform size were surface-sterilized, pre-germinated, and sown into cultivation bags filled with a perlite/vermiculite mixture (2:1, *v*/*v*), four plants per bag. Seedlings were raised in a light-controlled incubator (MGC-BP, Shanghai Yiheng Instruments, Shanghai, China) at 25 °C/22 °C (day/night) under a 16 h light/8 h dark photoperiod and a light intensity of 9000 lx, with Hoagland nutrient solution applied every 5–7 days. Once true leaves had emerged, healthy seedlings of comparable growth were selected for low-temperature treatment. Based on established cold-treatment protocols [2,25], plants assigned to the cold-treatment group were transferred to a growth chamber maintained at 4 °C to impose chilling stress.

To minimize the effects of light and circadian rhythms on starch metabolism, light intensity, photoperiod, and relative humidity were maintained consistently between the control and cold-treatment conditions. Control plants were maintained at 25 °C. Independent leaf samples assigned to the cold-treatment and control groups were collected separately at 0 h, and samples from both groups were subsequently collected at 1, 3, 6, 12, 24, and 48 h after treatment initiation. At each time point, the first and second fully expanded true leaves from the shoot apex were harvested. Three biological replicates were collected for each treatment–time combination, with each biological replicate consisting of pooled leaf tissue from three plants. Independent sets of plants were used for different biological replicates and sampling time points. All samples were immediately frozen in liquid nitrogen and stored at −80 °C until analysis.

### 4.2. Assay of β-Amylase Activity and Starch Content in Leaves

*S. tora* leaves were ground to a fine powder for enzyme assays. Approximately 0.1 g of fresh leaf tissue was ground into a fine powder and used for enzyme extraction. β-amylase activity was measured using a β-Amylase Activity Assay Kit (Cat. No. BC2045, Solarbio, Beijing, China) according to the manufacturer’s instructions. Absorbance was measured at 540 nm, and β-Amylase activity was calculated according to the formula provided by the manufacturer and expressed as units per gram fresh weight (U/g FW).

For starch determination, soluble sugars were first removed by washing with 80% ethanol in an 80 °C water bath. The pellet was gelatinized and hydrolyzed to glucose with the extraction reagent supplied in the Starch Content Assay Kit (colorimetric method, Sangon Biotech, Shanghai, China). Absorbance was read at 620 nm, and starch content was determined from a standard curve and expressed as mg per gram fresh weight (mg/g FW). All assays were run in three biological replicates.

### 4.3. Identification and Physicochemical Characterization of the BAM Gene Family

The whole-genome annotation files of *S. tora* were downloaded from NABIC (http://nabic.rda.go.kr/Species/Senna_tora2, accessed on 20 May 2026). BAM gene family data for *Arabidopsis* thaliana (AtBAMs) and *Oryza sativa* (OsBAMs) were obtained from TAIR (https://www.arabidopsis.org, accessed on 20 May 2026) and the Rice Genome Annotation Project website (http://rice.uga.edu, accessed on 20 May 2026), respectively. Genomic data for peanut (*A. hypogaea*) were retrieved from the PeanutBase database (https://www.peanutbase.org, accessed on 20 May 2026). Whole-genome sequence and annotation files for soybean (*G. max*), barrel medic (*M. truncatula*), garden pea (*P. sativum*), and adzuki bean (*V. angularis*) were downloaded from the Ensembl Plants database (https://plants.ensembl.org/index.html, accessed on 20 May 2026). Genomic data for licorice (*G. uralensis*) were obtained from the Genome Warehouse (GWH) of the National Genomics Data Center (NGDC) (https://ngdc.cncb.ac.cn/gwh, accessed on 20 May 2026). The genome assembly and GFF3 annotation for *Astragalus membranaceus* were downloaded from the *Astragalus membranaceus* Fuctional Genomics Database (AmDB; https://bioinformatics.nwnu.edu.cn/AmDB/download.php, accessed on 20 May 2026).

The hidden Markov model (HMM) profile of the glycoside hydrolase family 14 domain (Glyco_hydro_14; PF01373) was downloaded from the Pfam database [44] and used to search the predicted proteomes of the eight legume species using HMMER with default parameters [45]. For genes with multiple transcript isoforms, only the longest encoded protein was retained as the representative protein. Redundant sequences were removed, and the remaining candidates were further examined using SMART (http://smart.embl-heidelberg.de, accessed on 20 May 2026) and InterProScan (http://www.ebi.ac.uk/interpro, accessed on 20 May 2026) to confirm the presence of the conserved Glyco_hydro_14 domain [46,47]. The analysis recovered the same number of peanut BAM members as reported previously [23], and the same procedure was applied to the other seven legume species. The identified BAM genes were named according to their chromosomal positions. The nine *Arabidopsis* AtBAM and ten rice OsBAM proteins, retrieved from TAIR and RGAP, were not used as queries for this search; they were included only as reference sequences in the phylogenetic, gene structure and conserved motif analyses.

The protein length, molecular weight (MW), and theoretical isoelectric point (pI) of the 111 BAM proteins from the ten species examined were calculated using the ExPASy ProtParam tool (http://web.expasy.org/protparam, accessed on 20 May 2026). Their subcellular localization was predicted using BUSCA (http://busca.biocomp.unibo.it, accessed on 20 May 2026).

### 4.4. Phylogenetic Analysis, Gene Structure, and Conserved Motif Identification

Phylogenetic analyses were performed in MEGA 12 (http://www.megasoftware.net, accessed on 20 May 2026) using the full-length amino acid sequences of BAM proteins. The sequences were aligned using MUSCLE with default alignment parameters. Neighbor-Joining trees were constructed using the p-distance model, assuming uniform substitution rates among sites and applying pairwise deletion to gaps and missing data. Branch support was evaluated using 1000 bootstrap replicates. The phylogenetic tree of CBF/DREB1 proteins was constructed using the same procedure. Exon–intron structures were analyzed using TBtools-II (v2.472) [48]. Conserved motifs in BAM proteins were identified using the MEME online tool (http://meme-suite.org/tools/meme, accessed on 20 May 2026).

### 4.5. Identification and Phylogenetic Analysis of CBF/DREB1 Proteins

The six *Arabidopsis* CBF/DREB1 protein sequences, including AtCBF1 (AT4G25490), AtCBF2 (AT4G25470), AtCBF3 (AT4G25480), AtCBF4 (AT5G51990), AtDREB1E (AT1G63030), and AtDREB1F (AT1G12610), were used as queries to search the predicted *S. tora* proteome using BLASTP (version 2.16.0). Four non-redundant candidate CBF/DREB1 proteins were identified and designated StoCBF1–StoCBF4. For comparative phylogenetic analysis, the six *Arabidopsis* CBF/DREB1 proteins, 16 MtCBF proteins for *M. truncatula*, and 25 MsCBF proteins for *M. sativa* were obtained according to Cui et al. [49]. Among the 19 rice CBF genes reported by Cao et al. [50], ten proteins, OsCBF1–OsCBF10, containing a complete AP2 DNA-binding domain were retained for analysis, and proteins lacking a complete AP2 domain were excluded. The full-length amino acid sequences were used for phylogenetic analysis following the procedure described above.

### 4.6. Gene Duplication, Synteny and Sequence Similarity Analysis

Gene duplication events within each of the eight legume species, as well as syntenic relationships between *S. tora* and nine other species (including *A. thaliana* and *O. sativa*), were analyzed using the One Step MCScanX-Super Fast function in TBtools-II [48]. All results were visualized using the Advanced Circos tool. Local synteny maps were generated using the Find Path by Gene Pairs function in TBtools-II. Coding sequence (CDS) and protein sequence similarities were calculated using Clustal Omega (https://www.ebi.ac.uk/Tools/msa/clustalo, accessed on 20 May 2026) and visualized as heatmaps using the Heatmap Illustrator function in TBtools-II.

### 4.7. Expression Profiling of StoBAM Genes Across Tissues

To examine the tissue-specific expression patterns of *StoBAM* family members, publicly available RNA-seq data from *S. tora* root, leaf, early-stage seed, and late-stage seed samples were obtained from the NCBI Sequence Read Archive under study accession SRP159435 [51]. Read processing and quantification were carried out within the TBtools-II-RNAseq graphical interface. Raw reads were first assessed for sequence quality with FastQC, after which adapters and low-quality bases were trimmed using Trimmomatic. The cleaned reads of each sample were then pseudo-aligned to the *S. tora* reference transcriptome and quantified at the transcript level with Kallisto, yielding transcripts per million (TPM) values for all *StoBAM* members. To improve the resolution of the heatmap, TPM values were transformed to log2 (TPM + 1), and the resulting expression matrix was visualized in TBtools-II.

### 4.8. RNA Extraction and Quantitative Real-Time PCR (RT-qPCR)

Total RNA was isolated from *S. tora* leaves collected at each cold-treatment time point using an RNA extraction kit (TIANGEN, Beijing, China) according to the manufacturer’s protocol, with three biological replicates per treatment–time combination. RNA concentration and integrity were assessed using a NanoDrop ND-1000 spectrophotometer (NanoDrop, Wilmington, DE, USA) and 1% agarose gel electrophoresis. First-strand cDNA was synthesized from total RNA with SMARTScribe™ Reverse Transcriptase (Takara, Dalian, China) following the supplier’s instructions. RT-qPCR was performed using ChamQ Universal SYBR qPCR Master Mix (Vazyme, Nanjing, China) on an ABI QuantStudio 6 Flex real-time PCR system (Applied Biosystems, Foster City, CA, USA), with StoActin used as the internal reference gene for normalization. The thermal program comprised an initial denaturation at 95 °C for 30 s, followed by 40 cycles of 95 °C for 10 s and 60 °C for 30 s, with a final melting-curve step. Each reaction was run in three technical replicates. Relative expression levels of the target genes were calculated by the 2^−ΔΔCt^ method [52]. Gene-specific primers are listed in Table S2.

### 4.9. Yeast One-Hybrid (Y1H) Assay

To test whether StoCBF2 binds the promoters of *StoBAM6* and *StoBAM10*, a yeast one-hybrid (Y1H) assay was performed using a Y187-pHis2 kit (Coolaber, Beijing, China) according to the manufacturer’s instructions. Promoter fragments of 1978 bp for *StoBAM6* and 1906 bp for *StoBAM10* were amplified from *S. tora* genomic DNA and separately inserted into the pHis2 vector to generate *StoBAM6pro*-pHis2 and *StoBAM10pro*-pHis2, respectively. The promoter fragments (1978 bp for *StoBAM6* and 1906 bp for *StoBAM10*) correspond to the region of about 2 kb upstream of the translation start site, which is the same region analyzed for cis-regulatory elements in Section 2.6; the *StoBAM6* fragment contains the putative DRE-core motif. The exact fragment boundaries were determined by primer design: primers were placed to avoid repetitive sequences and to prevent primer-dimer formation, and the positions were also constrained by the restriction enzyme sites used for cloning into pHis2. As a result, the fragments are slightly shorter than 2000 bp and differ in length between the two genes. The full-length coding sequence of *StoCBF2* (Sto07g214110; 675 bp) was inserted into pGADT7 to generate *StoCBF2*-pGADT7. In this system, binding of the GAL4 activation domain–StoCBF2 fusion protein to the promoter fragment upstream of the *HIS3* reporter in the yeast nucleus activates *HIS3* expression, allowing growth on medium lacking histidine. To determine the appropriate 3-AT concentration for suppressing background HIS3 expression, yeast cells carrying each bait construct were separately grown on SD/-Trp/-His medium containing increasing concentrations of 3-AT. The concentrations tested were 0, 10, 20, 30 and 40 mM for *StoBAM6pro*-pHis2 and 0, 10, 20 and 30 mM for *StoBAM10pro*-pHis2. For the interaction assay, the corresponding bait and prey constructs were co-transformed into Y187 cells. Transformants were first selected on double-dropout medium (DDO; SD/-Leu/-Trp) and subsequently grown on triple-dropout medium (TDO; SD/-Trp/-Leu/-His) containing 40 mM 3-AT for *StoBAM6pro*-pHis2 or 30 mM 3-AT for *StoBAM10pro*-pHis2. The p53-His2 + p53-pGADT7 combination served as the positive control. p53-His2 + pGADT7, p53-His2 + StoCBF2-pGADT7, *StoBAM6pro*-pHis2 + pGADT7, and *StoBAM10pro*-pHis2 + pGADT7 were used as negative controls. Yeast cells were incubated at 28–30 °C for 48–72 h before growth was assessed. Primers used in this study are listed in Table S3.

### 4.10. Statistical Analysis

All physiological measurements (β-amylase activity, starch content) and RT-qPCR data were obtained from three independent biological replicates per time point and treatment group. Each biological replicate consisted of pooled tissue from three plants, and independent sets of plants were sampled for different biological replicates and time points. Separate biological samples were collected for the cold-treatment and control groups at 0 h. Data are presented as mean ± standard deviation (SD). For the expression analyses (Figure 7A,D and Figure 10), the effects of temperature treatment, sampling time and their interaction were evaluated using two-way analysis of variance (ANOVA) followed by Duncan’s multiple range test. Different lowercase letters indicate significant differences among treatment–time combinations at *p* < 0.05. Pearson’s correlation analyses were performed using paired measurements of *StoBAM6* or *StoBAM10* transcript abundance, β-amylase activity and starch content obtained from the same cold-treated biological samples. Each analysis included 21 paired observations from independently sampled plants, comprising seven sampling time points and three biological replicates per time point. Control samples were excluded from the correlation analyses. Statistical analyses were carried out in SPSS 26.0 (IBM, Armonk, NY, USA) and visualized using GraphPad Prism 9 (GraphPad Software, Boston, MA, USA).

## 5. Conclusions

In this study, we performed a comparative analysis of the *BAM* gene family across eight legume genomes. Within *S. tora*, *StoBAM6* and *StoBAM10* were identified as duplicated *AtBAM3*-like genes forming a collinear paralogous pair that is unlikely to have originated from the Papilionoideae-specific WGD. The two genes exhibited distinct cold-responsive expression patterns. *StoBAM6* was rapidly and persistently induced by cold, and its transcript abundance was positively associated with leaf β-amylase activity and negatively associated with starch content. In contrast, *StoBAM10* showed only a transient cold response, consistent with regulatory divergence between the two duplicated genes. *StoCBF2* was also strongly induced by cold, and the encoded StoCBF2 protein bound a *StoBAM6* promoter fragment harboring the DRE-core motif in yeast. These findings identify *StoBAM6* as a cold-responsive candidate associated with β-amylase activity and starch content and suggest that StoCBF2 may act as an upstream regulator of *StoBAM6*. Further genetic and in planta promoter assays are required to establish their roles in cold tolerance and to determine the functional significance of the divergence between *StoBAM6* and *StoBAM10*.

## Data Availability

The data presented in this study are available on request from the corresponding authors.

## Supplementary Materials

The following supporting information can be downloaded at https://www.mdpi.com/article/10.3390/plants15213407/s1, Figure S1: Phylogenetic relationships of CBF family members from *Senna tora*, *Oryza sativa*, *Medicago truncatula*, *Medicago sativa*, and *Arabidopsis thaliana*; Figure S2: Determination of the optimal 3-AT concentration for suppressing the self-activation activity of the *StoBAM6* promoter in yeast; Figure S3: Determination of the optimal 3-AT concentration for suppressing the self-activation activity of the *StoBAM10* promoter in yeast; Table S1: Classification and physicochemical properties of BAM proteins analyzed in this study; Table S2: Primers for RT-qPCR analysis; Table S3: Primers for yeast one-hybrid assays.
