# Supplementary material for: Genome-Wide Identification of the β-Amylase (BAM) Gene Family in Senna tora and Seven Other Legumes: Characterization of StoBAM6 as a Cold-Responsive Candidate Gene and a Potential Target of StoCBF2
Source: Plants (Basel). 2026 Nov 2;15(21):3407. doi: 10.3390/plants15213407 (PMC13765613; doi:10.3390/plants15213407)
Supplement: Supplementary file 1 [file plants-15-03407-s001.zip › plants-4664665-supplementary.pdf]

Supplementary Materials

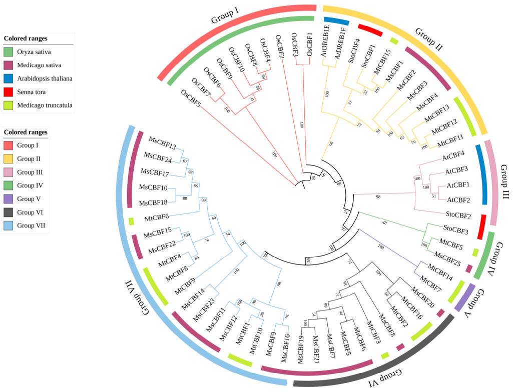

Figure S1. Phylogenetic relationships of CBF family members from *Senna tora*, *Oryza sativa*, *Medicago truncatula*, *Medicago sativa*, and *Arabidopsis thaliana*. Colored branches and outer arcs indicate the seven phylogenetic groups (I-VII), whereas inner colored arcs indicate species. The four *S. tora* members are marked by red arcs. Numbers at nodes indicate branch support values. StoCBF2 is placed in Group III together with AtCBF1, AtCBF2, AtCBF3, and AtCBF4.

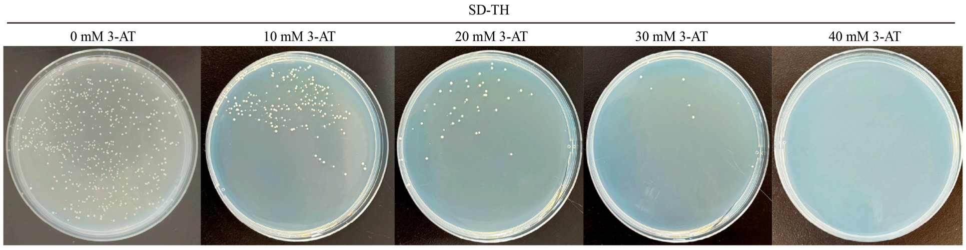

Figure S2. Determination of the optimal 3-AT concentration for suppressing the self-activation activity of the *StoBAM6* promoter in yeast. Yeast cells carrying the bait construct *StoBAM6pro*-pHis2 were plated on SD/-Trp/-His (SD-TH) selective medium supplemented with increasing concentrations of 3-amino-1,2,4-triazole (3-AT; 0, 10, 20, 30, and 40 mM) to evaluate background (leaky) expression of the HIS3 reporter gene. As the 3-AT concentration increased, the number of yeast colonies progressively decreased, and growth was essentially abolished at 40 mM 3-AT. These results indicated that the *StoBAM6* promoter exhibits weak self-activation activity on SD-TH medium, and that 40 mM 3-AT is sufficient to suppress this background growth. Accordingly, 40 mM 3-AT was selected as the working concentration for subsequent yeast one-hybrid (Y1H) one-to-one interaction assays.

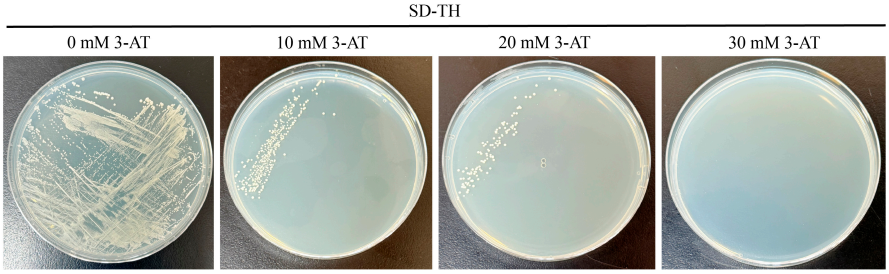

Figure S3. Determination of the optimal 3-AT concentration for suppressing the self-activation activity of the *StoBAM10* promoter in yeast. Yeast cells carrying the bait construct *StoBAM10pro*-

pHis2 were plated on SD/-Trp/-His (SD-TH) selective medium supplemented with increasing concentrations of 3-amino-1,2,4-triazole (3-AT; 0, 10, 20, and 30 mM) to evaluate background (leaky) expression of the HIS3 reporter gene. As the 3-AT concentration increased, the number of yeast colonies progressively decreased, and growth was essentially abolished at 30 mM 3-AT. These results indicated that the *StoBAM10* promoter exhibits weak self-activation activity on SD-TH medium, and that 30 mM 3-AT is sufficient to suppress this background growth. Accordingly, 30 mM 3-AT was selected as the working concentration for subsequent yeast one-hybrid (Y1H) one-to-one interaction assays.

Table S1. Classification and physicochemical properties of BAM proteins analyzed in this study.

Protein name	Group or Subgroup	Species	Gene ID	Protein Length (aa)	MW (kDa)	Theoretical pI	Predicted Subcellular Localization
AtBAM1	II-A	*Arabidopsis thaliana*	AT3G23920.1	575	63.76	5.7	chloroplast
AtBAM2	IV-A	*Arabidopsis thaliana*	AT4G00490.1	542	61.4	4.87	chloroplast thylakoid membrane
AtBAM3	II-B	*Arabidopsis thaliana*	AT4G17090.1	548	61.35	6.59	chloroplast
AtBAM4	III	*Arabidopsis thaliana*	AT5G55700.1	531	60.12	8.74	mitochondrion
AtBAM5	I	*Arabidopsis thaliana*	AT4G15210.1	498	56.06	5.2	extracellular space
AtBAM6	I	*Arabidopsis thaliana*	AT2G32290.1	577	66.57	6.33	chloroplast
AtBAM7	IV-A	*Arabidopsis thaliana*	AT2G45880.1	691	77.13	6.18	nucleus
AtBAM8	IV-B	*Arabidopsis thaliana*	AT5G45300.3	703	79.01	5.96	nucleus
AtBAM9	III	*Arabidopsis thaliana*	AT5G18670.1	536	58.28	5.51	chloroplast
OsBAM1	IV-B	*Oryza sativa*	LOC_Os02g03690.1	801	88.23	6.34	nucleus
OsBAM2	I	*Oryza sativa*	LOC_Os07g35940.1	488	55.12	5.24	extracellular space
OsBAM3	II-A	*Oryza sativa*	LOC_Os03g04770.1	557	60.76	5.53	mitochondrion
OsBAM4	III	*Oryza sativa*	LOC_Os07g47120.1	523	56.76	5.78	chloroplast
OsBAM5	III	*Oryza sativa*	LOC_Os03g22790.1	524	54.66	6	chloroplast
OsBAM6	IV-A	*Oryza sativa*	LOC_Os09g39570.1	533	59.71	5.64	chloroplast thylakoid membrane
OsBAM7	II-A	*Oryza sativa*	LOC_Os10g32810.1	535	57.95	6.74	chloroplast thylakoid membrane
OsBAM8	II-C	*Oryza sativa*	LOC_Os01g13550.1	566	61.69	9.19	chloroplast thylakoid membrane
OsBAM9	II-B	*Oryza sativa*	LOC_Os10g41550.1	544	59.14	6.31	chloroplast thylakoid membrane
OsBAM10	I	*Oryza sativa*	LOC_Os07g35880.1	600	66.71	5.22	mitochondrion
StoBAM1	II-A	*Senna tora*	Sto03g058680	597	65.92	5.93	chloroplast thylakoid membrane
StoBAM2	III	*Senna tora*	Sto03g063580	532	58.47	5.95	chloroplast
StoBAM3	IV-A	*Senna tora*	Sto06g182230	851	94.75	8.77	nucleus
StoBAM4	IV-A	*Senna tora*	Sto06g182240	263	29.63	4.6	nucleus
StoBAM5	II-C	*Senna tora*	Sto06g192750	678	77.36	8.79	mitochondrion
StoBAM6	II-B	*Senna tora*	Sto06g196680	542	61.09	8.55	chloroplast
StoBAM7	II-C	*Senna tora*	Sto07g218100	323	36.43	10.1	nucleus
StoBAM8	III	*Senna tora*	Sto10g346660	554	61.68	5.92	nucleus
StoBAM9	IV-B	*Senna tora*	Sto11g362670	804	91.14	5.9	nucleus
StoBAM10	II-B	*Senna tora*	Sto11g367770	624	70.45	7.28	chloroplast thylakoid membrane
GmBAM1	I	*Glycine max*	transcript:KRH56074	496	56.07	5.33	extracellular space
GmBAM2	IV-B	*Glycine max*	transcript:KRH58233	222	26.07	6.38	endomembrane system
GmBAM3	II-C	*Glycine max*	transcript:KRH59949	557	63.07	9.18	chloroplast thylakoid membrane
GmBAM4	IV-A	*Glycine max*	transcript:KRH40463	705	78.84	5.16	nucleus
GmBAM5	III	*Glycine max*	transcript:KRH11269	536	58.75	5.83	chloroplast

GmBAM6	IV-B	*Glycine max*	transcript:KRH76424	656	74.40	5.76	nucleus
GmBAM7	I	*Glycine max*	transcript:RCW19114	460	52.51	8.91	chloroplast
GmBAM8	II-B	*Glycine max*	transcript:KRH77272	548	61.79	8.53	chloroplast
GmBAM9	I	*Glycine max*	transcript:KRH26846	601	68.55	6.31	chloroplast
GmBAM10	I	*Glycine max*	transcript:KRH22506	573	66.01	8.52	chloroplast
GmBAM11	IV-A	*Glycine max*	transcript:KRH00731	704	78.84	5.31	nucleus
GmBAM12	I	*Glycine max*	transcript:KRH25439	496	56.03	5.26	extracellular space
GmBAM13	III	*Glycine max*	transcript:KRH21019	536	58.78	5.64	nucleus
GmBAM14	II-B	*Glycine max*	transcript:KRH57551	547	61.69	8.03	chloroplast
GmBAM15	II-C	*Glycine max*	transcript:KRH41367	553	62.59	8.72	chloroplast thylakoid membrane
GmBAM16	II-B	*Glycine max*	transcript:KRH04262	540	60.54	7.66	chloroplast thylakoid membrane
GmBAM17	IV-B	*Glycine max*	transcript:KRH39743	654	74.12	5.87	nucleus
GmBAM18	II-A	*Glycine max*	transcript:KRH38943	569	62.98	5.75	mitochondrion
GmBAM19	II-B	*Glycine max*	transcript:KRH28213	554	62.49	8.69	chloroplast
GmBAM20	II-A	*Glycine max*	transcript:KRH09479	570	62.95	5.76	chloroplast thylakoid membrane
AhBAM1	II-B	*Arachis hypogaea*	arahy.Tifrunner.gnm2.ann1.Arahy.3VMA9Y.1	575	64.65	8.98	chloroplast
AhBAM2	I	*Arachis hypogaea*	arahy.Tifrunner.gnm2.ann1.Arahy.NQ88PQ.2	559	62.97	5.83	chloroplast
AhBAM3	II-C	*Arachis hypogaea*	arahy.Tifrunner.gnm2.ann1.Arahy.U96YPL.1	577	65.15	8.86	chloroplast thylakoid membrane
AhBAM4	III	*Arachis hypogaea*	arahy.Tifrunner.gnm2.ann1.Arahy.I36EUT.1	533	58.37	6.07	nucleus
AhBAM5	IV-A	*Arachis hypogaea*	arahy.Tifrunner.gnm2.ann1.Arahy.UANN5Y.1	1144	128.50	5.86	plasma membrane
AhBAM6	IV-B	*Arachis hypogaea*	arahy.Tifrunner.gnm2.ann1.Arahy.K0N155.1	647	73.09	6.01	nucleus
AhBAM7	III	*Arachis hypogaea*	arahy.Tifrunner.gnm2.ann1.Arahy.68PBWJ.1	229	25.22	6.64	extracellular space
AhBAM8	II-A	*Arachis hypogaea*	arahy.Tifrunner.gnm2.ann1.Arahy.4Y2508.1	585	64.58	6.27	chloroplast thylakoid membrane
AhBAM9	I	*Arachis hypogaea*	arahy.Tifrunner.gnm2.ann1.Arahy.C3I2PC.1	586	66.25	7.58	cytoplasm
AhBAM10	II-B	*Arachis hypogaea*	arahy.Tifrunner.gnm2.ann1.Arahy.34GL3D.1	544	61.00	8.69	chloroplast
AhBAM11	I	*Arachis hypogaea*	arahy.Tifrunner.gnm2.ann1.Arahy.0V89VS.2	441	49.76	5.48	extracellular space
AhBAM12	II-C	*Arachis hypogaea*	arahy.Tifrunner.gnm2.ann1.Arahy.ABG1ZW.1	559	63.11	8.67	mitochondrion

AhBAM13	III	*Arachis hypogaea*	arahy.Tifrunner.gnm2.ann1.Arahy.60DV95.1	533	58.33	5.94	nucleus
AhBAM14	IV-A	*Arachis hypogaea*	arahy.Tifrunner.gnm2.ann1.Arahy.M409IX.1	1109	124.44	5.72	nucleus
AhBAM15	IV-B	*Arachis hypogaea*	arahy.Tifrunner.gnm2.ann1.Arahy.Y0TSMC.1	647	73.09	6.01	nucleus
AhBAM16	III	*Arachis hypogaea*	arahy.Tifrunner.gnm2.ann1.Arahy.JJTS0M.1	482	53.30	6.22	chloroplast
AhBAM17	II-A	*Arachis hypogaea*	arahy.Tifrunner.gnm2.ann1.Arahy.TP5BL7.1	585	64.67	6.07	chloroplast thylakoid membrane
AhBAM18	I	*Arachis hypogaea*	arahy.Tifrunner.gnm2.ann1.Arahy.Y6Z03A.1	606	68.61	6.93	endomembrane system
MtBAM1	III	*Medicago truncatula*	Medtr2g020240.1.JCVIMt4.0v1	535	58.68	6.32	nucleus
MtBAM2	I	*Medicago truncatula*	Medtr2g089030.1.JCVIMt4.0v1	590	67.55	6.04	chloroplast
MtBAM3	I	*Medicago truncatula*	Medtr4g045667.1.JCVIMt4.0v1	496	56.21	5.16	extracellular space
MtBAM4	II-B	*Medicago truncatula*	Medtr4g099110.1.JCVIMt4.0v1	541	60.77	7.27	chloroplast
MtBAM5	II-B	*Medicago truncatula*	Medtr5g013620.1.JCVIMt4.0v1	543	60.78	6.58	chloroplast
MtBAM6	II-B	*Medicago truncatula*	Medtr5g013640.1.JCVIMt4.0v1	543	60.68	7.62	chloroplast
MtBAM7	IV-B	*Medicago truncatula*	Medtr5g026210.1.JCVIMt4.0v1	650	73.86	5.53	nucleus
MtBAM8	II-A	*Medicago truncatula*	Medtr6g477780.1.JCVIMt4.0v1	384	44.05	5.3	cytoplasm
MtBAM9	IV-A	*Medicago truncatula*	Medtr7g077410.1.JCVIMt4.0v1	702	78.53	5.35	nucleus
MtBAM10	II-C	*Medicago truncatula*	Medtr8g099775.1.JCVIMt4.0v1	545	62.06	8.78	chloroplast thylakoid membrane
GurBAM1	II-B	*Glycyrrhiza uralensis*	GUL01G003721.mRNA1	545	61.37	8.3	chloroplast
GurBAM2	I	*Glycyrrhiza uralensis*	GUL01G004110.mRNA1	570	65.17	5.22	chloroplast
GurBAM3	IV-A	*Glycyrrhiza uralensis*	GUL02G002721.mRNA1	704	79.14	5.37	nucleus
GurBAM4	I	*Glycyrrhiza uralensis*	GUL03G004157.mRNA1	496	56.45	4.91	extracellular space
GurBAM5	II-C	*Glycyrrhiza uralensis*	GUL03G005194.mRNA1	554	63.35	9.22	chloroplast thylakoid membrane
GurBAM6	IV-B	*Glycyrrhiza uralensis*	GUL06G003455.mRNA1	657	74.07	5.87	nucleus
GurBAM7	II-B	*Glycyrrhiza uralensis*	GUL06G004054.mRNA1	592	66.14	8.12	chloroplast
GurBAM8	I	*Glycyrrhiza uralensis*	GUL07G002656.mRNA1	588	67.39	5.83	chloroplast
GurBAM9	III	*Glycyrrhiza uralensis*	GUL07G004027.mRNA1	532	58.69	5.95	chloroplast
GurBAM10	II-A	*Glycyrrhiza uralensis*	GUL08G002276.mRNA1	571	63.31	6.01	chloroplast thylakoid membrane
AmBAM1	IV-A	*Astragalus membranaceus*	Am01G023860.1	661	73.91	6.4	nucleus
AmBAM2	IV-B	*Astragalus membranaceus*	Am01G023980.1	1332	149.34	6.61	plasma membrane

AmBAM3	IV-A	*Astragalus membra-naceus*	Am01G038300.1	534	60.34	5.05	cytoplasm
AmBAM4	II-B	*Astragalus membra-naceus*	Am04G004260.1	549	61.49	8.56	chloroplast
AmBAM5	III	*Astragalus membra-naceus*	Am04G009710.1	513	56.43	7.95	chloroplast thylakoid membrane
AmBAM6	II-A	*Astragalus membra-naceus*	Am05G034100.1	573	63.62	5.91	cytoplasm
AmBAM7	III	*Astragalus membra-naceus*	Am06G006220.1	541	59.55	5.99	chloroplast
AmBAM8	I	*Astragalus membra-naceus*	Am07G013040.1	573	63.98	5.74	nucleus
PsBAM1	II-B	*Pisum sativum*	transcript:Psat1g138200.1	546	61.20	6.33	chloroplast
PsBAM2	III	*Pisum sativum*	transcript:Psat1g187120.1	539	59.41	7.24	nucleus
PsBAM3	IV-B	*Pisum sativum*	transcript:Psat2g144320.1	649	73.75	5.68	nucleus
PsBAM4	IV-A	*Pisum sativum*	transcript:Psat3g108680.1	799	89.43	4.92	nucleus
PsBAM5	II-B	*Pisum sativum*	transcript:Psat4g076800.1	459	51.44	6.71	cytoplasm
PsBAM6	I	*Pisum sativum*	transcript:Psat5g265560.1	588	67.44	6.15	chloroplast
PsBAM7	II-C	*Pisum sativum*	transcript:Psat7g023600.1	554	63.51	9.14	chloroplast thylakoid membrane
PsBAM8	I	*Pisum sativum*	transcript:Psat7g197880.1	528	59.91	5.29	chloroplast
VaBAM1	II-B	*Vigna angularis*	transcript:KOM32504	548	61.43	8.59	chloroplast
VaBAM2	I	*Vigna angularis*	transcript:KOM43969	510	58.81	6.91	extracellular space
VaBAM3	I	*Vigna angularis*	transcript:KOM43972	584	67.60	8.71	chloroplast
VaBAM4	II-B	*Vigna angularis*	transcript:KOM44868	555	62.47	8.82	chloroplast
VaBAM5	IV-B	*Vigna angularis*	transcript:KOM45458	662	75.17	6.04	nucleus
VaBAM6	I	*Vigna angularis*	transcript:KOM50432	496	56.23	5.73	extracellular space
VaBAM7	III	*Vigna angularis*	transcript:KOM53529	532	58.48	5.66	nucleus
VaBAM8	II-A	*Vigna angularis*	transcript:KOM54558	561	62.11	5.77	chloroplast thylakoid membrane

Table S2. Primers for RT-qPCR analysis.

Gene	Gene ID	Forward Primer Sequence	Reverse Primer Sequence	Size (bp)
StoBAM6	Sto06g196680	GACTCCTGCAGCATCCCTCTAC	GGCAATGAATCGCAGCCTAATGAG	128
StoBAM10	Sto11g367770	ACTGGGAAGCCTATGTTGAGCTTG	GCAGAGGAATACTGCAGGAGTCG	120
StoCBF1	Sto06g194850	GATGAGCCTTTGAGGTCTCCGTT	GCTGCTAGAGCTTCAGTACACTCA	92
StoCBF2	Sto07g214110	GCTGGATAGTATGGCTGAGGTTCA	AGGATACCTCTAGAAGGCGTCCA	98
StoCBF3	Sto10g342150	ATGTTGACAGAGCCTCCCAACC	GAATCTCGGCCTCAGTTCGCA	111
StoCBF4	Sto11g369450	AACTTTGCGGACTCGGCTTGT	CGTCGTTCTTGGGGTCGTGAAA	118
StoActin	Sto08g277530	TCCTCTTAATCCCAAGGCTAACCG	ACCAGAGTCCAACACAATACCAGT	119

Table S3. Primers for yeast one-hybrid assays.

Construct	Enzyme Cleavage Site	Primer Sequence	PCR Product (bp)
StoBAM6pro-pHis2	*Sac*I<br>*Xba*I	For: *CGCGGAGCTC*AATACTTCCTCCTTCCTTCTCATGTTCTC<br>Rev: *GCGCTCTAGA*GATTGTGTTTGCAGTGGAAGGTAAGG	1998
StoBAM10pro-pHis2	*Sac*I<br>*Xba*I	For: *CGCGGAGCTC*TCTAACCCTACATTTTTAACTCAGTTTCTTGTG<br>Rev: *GCGCTCTAGA*TTCTTCTCGTGTAGTTGTGTTTTGCAG	1926
StoCBF2-pGADT7-Rec2	*Eco*RI<br>*Bam*HI	For: *CCGGGAATTC*ATGGGGAATATTACTAATGAAGTT-GCTTCTTCTC<br>Rev: *CGCGGGATCC*CTACGACTGCTCCTCCTCGTACATTTCTG	695
